# Soy-Based Therapeutic Baby Formulas: Testable Hypotheses Regarding the Pros and Cons

**DOI:** 10.3389/fnut.2016.00059

**Published:** 2017-01-18

**Authors:** Cara J. Westmark

**Affiliations:** ^1^Department of Neurology, University of Wisconsin, Madison, WI, USA

**Keywords:** soy-based infant formula, phytoestrogens, glyphosate, genetically modified, seizures, necrotizing enterocolitis

## Abstract

Soy-based infant formulas have been consumed in the United States since 1909, and currently constitute a significant portion of the infant formula market. There are efforts underway to generate genetically modified soybeans that produce therapeutic agents of interest with the intent to deliver those agents in a soy-based infant formula platform. The threefold purpose of this review article is to first discuss the pros and cons of soy-based infant formulas, then present testable hypotheses to discern the suitability of a soy platform for drug delivery in babies, and finally start a discussion to inform public policy on this important area of infant nutrition.

## Introduction

Soy-based infant formulas (SIFs) have been in use in the United States for over a century. Proponents of SIF argue that the long time and widespread use of SIF in the general population without documented adverse health effects proves they are a safe, healthy, and economical alternative for babies with cow milk allergies, lactose intolerance, galactosemia, gastrointestinal issues, or vegetarian preference. Opponents of SIF counter that the safety of SIF has not been rigorously tested, particularly health effects associated with the phytoestrogens and agrochemicals in soy products. Recently, an article entitled, “Transgenic Soybean Production of Bioactive Human Epidermal Growth Factor (EGF),” was published in *PLoS One* by the Herman Laboratory at the University of Arizona. The authors conclude that soybean seeds can be employed as biofactories for the production of therapeutic agents such as EGF with delivery in a soymilk platform. Our goals in this article are to review the available evidence regarding the safety and efficacy of SIF, present testable hypothesis that address the adverse arguments, and thus provide a framework for a public policy debate regarding the feasibility and bioethics of developing therapeutic SIF.

## The Evidence Supporting Soy-Based Therapeutic Infant Formulas

Three main arguments in support of developing therapeutic SIF are as follows: (1) SIFs have been safely used for over a century with minimal documented adverse health effects, (2) soy protein is associated with many health benefits in adults, and (3) soybeans are an economical means to generate and deliver therapeutics. First, we will review the literature in regard to randomized controlled trials (RCT) with SIF, then we will discuss soy-associated health benefits, and finally, we will consider the biological and economic advantages of utilizing SIF as a drug delivery platform.

In terms of documented health effects, RCT are the gold standard in testing the safety and efficacy of medical interventions. RCT are carefully planned clinical studies testing a treatment or exposure in patients. The methodologies are designed to reduce bias and systematic errors and provide sound evidence of cause and effect. Thus, we reviewed the literature in regard to RCT with SIF. First, we searched PubMed with the keywords “Soy” AND “Infant” AND “Formula” and the filters “From 2005/01/01 to 2016/07/19” AND “Clinical Trial.” Twenty-eight articles were returned (Table 1), of which 27 were available in English. Eleven of these articles regarded a side-by-side comparison of the effects of SIF with breast milk and/or cow milk-based formula on infant health (reviewed below). The remaining 16 SIF RCT are reviewed in the Data Sheet S1 in Supplementary Material.

**Table 1:** **PubMed literature search with keywords “Soy” AND “Infant” AND “Formula” and the Filters “From 2005/01/01 to 2016/07/19” AND “Clinical Trial**.”

1.	Soy protein-based infant formulas with supplemental fructooligosaccharides: gastrointestinal tolerance and hydration status in newborn infants	Lasekan et al. (1)
2.	Compared with feeding infants breast milk or cow milk formula, soy formula feeding does not affect subsequent reproductive organ size at 5 years of age	Andres et al. (2)
3.	Lactose-free milk or soy-based formulas do not improve caregivers’ distress or perceptions of difficult infant behavior	Sherman et al. (3)
4.	The use of linear programing to determine whether a formulated complementary food product can ensure adequate nutrients for 6- to 11-month-old Cambodian infants	Skau et al. (4)
5.	Formula selection for management of children with cow’s milk allergy (CMA) influences the rate of acquisition of tolerance: a prospective multicenter study	Berni Canani et al. (5)
6.	Body fat and bone mineral content of infants fed breast milk, cow’s milk formula, or soy formula during the first year of life	Andres et al. (6)
7.	Developmental status of 1-year-old infants fed breast milk, cow’s milk formula, or soy formula	Andres et al. (7)
8.	Evaluation of therapeutic effects of three hypoallergenic formula in infants with cow’s milk protein allergy	Yan et al. (8) (in Chinese)
9.	Soy isoflavone phase II metabolism differs between rodents and humans: implications for the effect on breast cancer risk	Setchell et al. (9)
10.	Effect of a partially hydrolyzed whey infant formula at weaning on risk of allergic disease in high-risk children: a randomized controlled trial	Lowe et al. (10)
11.	Effect of feeding a formula supplemented with long-chain polyunsaturated fatty acids at 14 weeks improves the *ex vivo* response to a mitogen and reduces the response to soy protein in infants at low risk for allergy	Field et al. (11)
12.	The effect of partially hyrdrolysed formula based on rice protein in the treatment of infants with cow’s milk protein allergy	Reche et al. (12)
13.	Effect of milk-based infant formula and soy-based infant formula (SIF) on *in situ* demineralization of human primary enamel	de Mazer Papa et al. (13)
14.	Impact of dietary regimen on the duration of CMA: a random allocation study	Terracciano et al. (14)
15.	Infant formula promotes bone growth in neonatal piglets by enhancing osteoblastogenesis through bone morphogenic protein signaling	Chen et al. (15)
16.	Comparison of yogurt, soybean, casein, and amino-acid-based diets in children with persistent diarrhea	de Mattos et al. (16)
17.	Early infant diet and the omega-3 fatty acid docosahexanenoic acid (DHA): effects on resting cardiovascular activity and behavioral development during the first half-year of life	Pivik et al. (17)
18.	Clinical response to two commonly used switch formulas occurs within 1 day	Berseth et al. (18)
19.	Comparisons of a chicken-based formula with soy-based formula in infants with CMA	Jirapinyo et al. (19)
20.	SIF supplemented with DHA and ARA supports growth and increases circulating levels of these fatty acids in infants	Hoffman et al. (20)
21.	The effect of early nutritional supplementation with a mixture of probiotic, prebiotic, fiber, and micronutrients in infants with acute diarrhea in Indonesia	Agustina et al. (21)
22.	Growth of infants with immunoglobulin E (IgE)-mediated CMA fed different formulas in the complementary feeding period	Agostoni et al. (22)
23.	Palatability of hydrolyzates and other substitution formulas for CMA children: a comparative study of taste, smell, and texture evaluated by healthy volunteers	Pedrosa et al. (23)
24.	A hydrolyzed rice-based formula is tolerated by children with CMA: a multicenter study	Fiocchi et al. (24)
25.	Decreased regurgitation with a soy formula containing added soy fiber	Ostrom et al. (25)
26.	Feeding a soy formula to children with CMA: the development of IgE-mediated allergy to soy and peanuts	Klemola et al. (26)
27.	The almond milk: a new approach to the management of CMA/intolerance in infants	Salpietro et al. (27)
28.	A follow-up study of nutrient intake, nutritional status, and growth in infants with CMA fed either soy formula or an extensively hydrolyzed whey formula	Seppo et al. (28)

### SIF RCT Literature Review 2005–2016

The Andres study (2) was a prospective, longitudinal study in children from the Beginnings Study who were recruited from the Central Arkansas region between ages 1 and 2 months of age and were tested at 5 years of age for reproductive organ volume and structural characteristics in response to infant diet. The population included 101 children (50 boys and 51 girls) aged 5 years who were breast-fed (*n* = 35) or fed cow milk formula (*n* = 32) or SIF (*n* = 34) as infants. The independent variable was infant diet, and the dependent variables were breast bud, uterus, ovaries, prostate, and testes volumes and characteristics as assessed by ultrasonography. The outcome in both genders was no significant difference in reproductive organ volume or structural characteristics.

The Sherman study (3) was a double-blind, multicenter, parallel-group, randomized study in infants to assess formula change on fussiness and caregiver distress. The study population included dyads of infants (2–12 weeks of age; mean age 4.97 weeks) and female caregivers. The infants had been fed a milk-based formula containing lactose for at least 5 days as the sole diet at the time of enrollment and were experiencing common feeding problems (i.e., fussiness/crying/cramping, gas, or diarrhea). Infants were randomized to receive lactose-free milk-based formula (*n* = 96), lactose-free SIF (*n* = 97), or milk-based, lactose-containing formula (*n* = 103) as the sole diet for 14 days. The independent variable was formula and the dependent variables were answers to the Infant Characteristics Questionnaire and measures of caregiver distress. The outcome was no significant benefit in reducing infant and maternal distress in response to milk-based or SIF lactose-free formulas compared with a lactose-containing milk-based formula.

The Berni Canani study (5) was a prospective, open, non-randomized evaluation over a 12-month period of the effect of different dietary management strategies on the rate of acquisition of tolerance in otherwise healthy children with CMA. The population included 260 male and female children aged 1–12 months. The study population was divided into five formula groups: (1) extensively hydrolyzed casein formula (EHCF), (2) EHCF + *Lactobacillus rhamnosus* GG, (3) hydrolyzed rice formula, (4) SIF, and (5) amino acid-based formula. The independent variable was formula choice and the dependent variable was tolerance to cow’s milk. The outcome was the rate of children acquiring oral tolerance after 12 months was significantly higher in groups receiving EHCF or EHCF + *L. rhamnosus* GG.

The Andres study (6) was a 12-month longitudinal study to characterize growth, fat mass, free fat mass, and bone mineral content (BMC) in healthy infants fed breast milk versus cow’s milk formula or SIF during the first year of life. The population included 344 infants aged 3, 6, 9, and 12 months, both male and female (the Beginnings Study). The ethnicity was 9.7% African-American, 84.5% Caucasian, and 5.8% unknown. Socioeconomic status (SES) was 46.8 ± 11.6 on Hollingshead Four-Factor Index. Growth was evaluated using standard anthropometric techniques and body composition was assessed using dual-energy X-ray absorption. Mixed-effects models with repeated measures were used to adjust for race, SES, gestational age, birth weight, birth length, sex, age, and diet history. The independent variable was formula choice, and the dependent variables were body composition and BMC. The outcome was that infants fed breast milk had higher fat mass at 3 months and lower free fat mass at 6–12 months. Infants fed SIF had greater free fat mass at age 6 and 9 months compared to cow milk formula. BMC was higher in infants fed breast milk and lower in infants fed SIF at 3 months. BMC was higher in infants fed SIF at 12 months. Body composition data showed that soy-fed infants were significantly leaner during the first 6 months than breast milk or cow milk fed as indicated by greater total free fat mass.

The Andres study (7) was a longitudinal study over 12 months to assess developmental status in healthy infants enrolled in the Beginnings Study between 2002 and 2010 and fed breast milk versus cow’s milk formula versus SIF during the first year of life. The population included 391 infants aged 1 year, both male and female. The ethnicity was 91% Caucasian, 4% African-American, and 5% others. The SES was 45.6 (10.6)–49.8 (11.0) on the Hollingshead scale. Exclusion criteria included a change of formula after 2 months of age and before 12 months, complementary foods before 4 months and/or body weight <5 kg at 3 months. Diet decisions were made before enrollment in the study. Healthy infants were assessed longitudinally at ages 3, 6, 9, and 12 months using Bayley Scales of Infant Development and the Preschool Language Scale-3. Mixed-effects models were used for statistical analyses to adjust for SES, mother’s age and intelligence quotient, gestational age, gender, birth weight, head circumference, race, age, and diet history. The independent variable was choice of formula, and the dependent variables were developmental status and anthropometric measures. The outcome was that infants fed SIF scored within normal limits on standardized developmental testing and did not differ from infants fed cow milk-based formula. Breast-fed infants had a slight advantage on cognitive development compared with formula fed.

The Lowe study (10) was a single-blind (patient) randomized control trial over a 7-year period comparing allergic outcomes between infants fed a conventional cow’s milk formula, a partially hydrolyzed whey formula, and SIF. The population included 620 male and female infants who were enrolled before birth and had a family history of allergic disease. Mothers were encouraged to initiate and maintain breast-feeding for at least 6 months. Study formulas were introduced at cessation of breast-feeding. Skin prick tests to six common allergens were performed at 6, 12, and 24 months. The independent variable was formula type (partially hydrolyzed whey formula versus soy versus casein), and the dependent variables were allergic manifestations (eczema and food reactions). The outcome was there was no evidence that infants randomized to the partially hydrolyzed whey formula or SIF were at lower risk of allergic manifestations in infancy compared to conventional formula. The primary outcome was allergic manifestation. The secondary outcomes were individual incidence of eczema and food reactions reported in the first 2 years; skin prick test reactivity at 6, 12, and 24 months; and prevalence of eczema, asthma, and allergic rhinitis in the first 2 years as well as at ages 6 and 7 years.

The Terracciano study (14) was a randomized trial to increase cow milk tolerance in CMA infants by treating with hydrolyzed rice formula or extensively hydrolyzed soy formula. Male and female infants aged 14.1 ± 8.6 months (*n* = 72) were studied over 26 months. The independent variable was diet (rice versus soy versus cow milk diets), and the dependent variable was tolerance to CMA. This was an intention-to-treat, randomized study. The symptomatic formula patients from the Milan Cow’s Milk Allergy Cohort were randomly switched to treatment groups (hydrolyzed rice formula, EHCF, and extensively hydrolyzed soy formula). Oral food challenges were conducted with follow-up visits every 3 months. Statistics included Kaplan–Meier curves after stratification for dietary option. The Cox model was used to estimate hazard ratios. The outcome was 51 children reached tolerance at a mean of 34.1 ± 15.2 months. Both rice and soy diets reduced the duration of CMA. In children who were not co-sensitized to soy, the duration of CMA was more strongly impacted by diet.

The Chen study (15) examined the effects of formula feeding relative to breast-feeding on bone in the neonate pig. Male and female newborn pigs (*n* = 30) were studied for 21 and 35 days. The independent variable was diet (SIF versus cow milk-based formula), and the dependent variables were bone mineral density and bone markers. The study employed peripheral quantitative computed tomography scans and histomorphometric analysis. The outcome was piglets fed either soy- or cow milk-based formula had greater bone mineral density than those breast-fed. Osteoblast numbers and bone formation rate were greater at postnatal day 35 (P35) with SIF. Osteoclast numbers were lower in both cow milk based and SIF fed than in breast-fed subjects. Bone formation markers were greater and bone resorption markers were lower in cow milk and soy groups than in the breast-fed. Formula feeding promoted bone growth compared with breast-fed.

The Pivik study (17) evaluated variations in resting heart rate measures during the first half-year of life in healthy, full-term infants who were either breast-fed, fed milk-based formula, or fed SIF ± docosahexanenoic acid (DHA) supplementation. The study population was drawn from the Beginning Study and included 64 male infants aged 2 months. The educational levels were given for both parents and were the lowest in mothers using SIF without DHA. The independent variable was formula type, and the dependent variables were anthropometrics and heart rate. Infants remained on parent-selected formula throughout study. Four diet groups included breast-fed (*n* = 20), milk formula (*n* = 21), SIF with DHA (*n* = 16), and SIF without DHA (*n* = 7). The outcome was DHA-deficient SIF resulted in higher heart rate and lower values for heart rate variability measures indicating decreased parasympathetic tone. Effects appeared at 4 months and continued for the remainder of study period. Later work by the same group found vaginal tone stability between infancy and 2 years emerged later in SIF-fed infants compared to breast and cow milk formula fed (29).

The Berseth study (18) was a multicenter (20 sites), double-blind, randomized, parallel, prospective 28-day feeding trial assessing body weight, infant formula tolerance and fussiness/gas/spit-up/crying in response to SIF and partially hydrolyzed formula. The study population included 159 fussy, male and female infants aged 7–63 days. The independent variable was diet, and the dependent variables were fussiness, crying, gas, spit-up, bowel movements and consistency, diarrhea, constipation, and results of the Infant Characteristic Questionnaire. Subjects were randomized to receive SIF or partially hydrolyzed formula with cow milk protein/low lactose. Measurements included body weight and infant formula tolerance. The outcome was a significant reduction in mean scores of fussiness, gas, spit-up, and crying compared to baseline in infants receiving soy or partially hydrolyzed formula within 1 day of formula intake. The improvement in symptoms remained constant through day 28. The soy group had firmer stools. Partially hydrolyzed formula, which has a protein profile closer to breast milk, improved formula intolerance as well as SIF.

The Agostoni study (22) investigated whether the type of milk in the complementary feeding period (6–12 months of age) was associated with growth. The study population included 160 male and female infants aged 6–12 months with immunoglobulin E (IgE)-mediated CMA and breast-fed for at least 4 months with progressive weaning in the 5- to 6-month period. The study length was 12 months. Subjects were randomly assigned to one of three special formulas versus controls that were breast-fed up to 12 months. Weight for age, length for age, and weight for length *z*-scores were measured at 6, 9, and 12 months of age. Standard statistical methods were used including ANOVA, chi-squared, and Fisher tests. The independent variable was formula type (soy, casein hydrolyzate, rice hydrolyzate), and the dependent variables were growth parameters. The outcome was impaired growth at 6 months of age in infants with CMA. The infants fed hydrolyzed products exhibited a trend for higher weight for age scores in the 6- to 12-month period.

**Figure 1:**
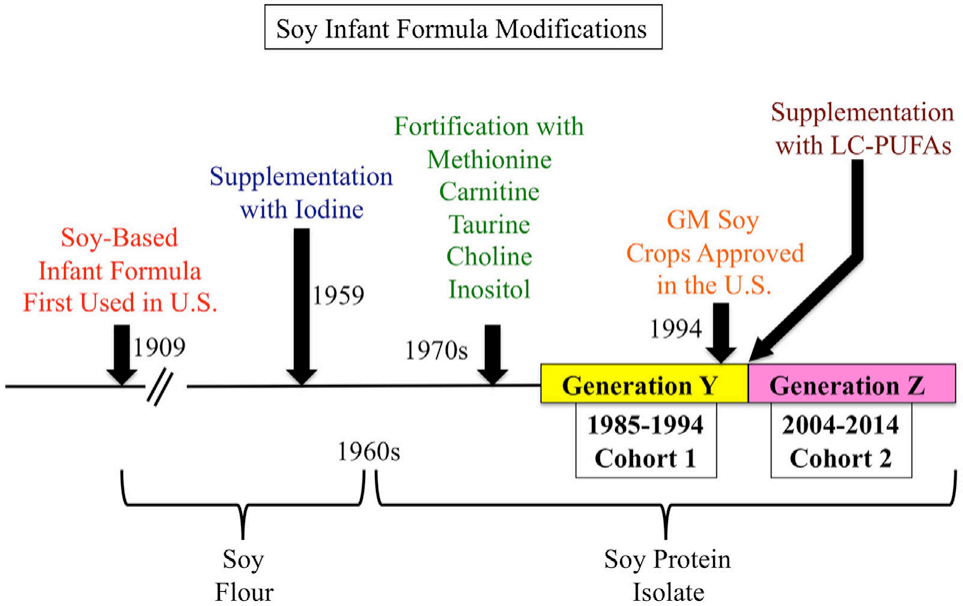
**History of soy-based infant formula (SIF)**. SIFs were first used in the United States in 1909 and were based on soy flour. Since then, SIFs have undergone multiple refinements. In 1959, SIFs were supplemented with iodine to prevent goiter. In the 1960s, soy flour was replaced with soy protein isolate to ease digestion and formulate a more balanced and higher concentration of essential amino acids. In the 1970s, SIFs were fortified with methionine, carnitine, taurine, choline, and inositol. In 1994, genetically modified soybeans were approved for agriculture in the United States. And about the year 2000, SIF were supplemented with long-chain polyunsaturated fatty acids.

In summary, infant formula can be switched for many reasons such as intolerance, food allergies, fussiness, and regurgitation. Findings from individual RCT indicate that SIF (1) does not induce tolerance to cow milk protein as quickly as EHCF and EHCF + *L. rhamnosus* GG, (2) alters growth metrics such that infants are leaner during the first 6 months with higher BMC at 12 months, and (3) does not affect cognitive ability in healthy infants. The period chosen for this data analysis (2005–2016) occurred after many manufacturing, supplementation, and agricultural practices were altered that could affect the nutrient content and bioactivity of SIF (Figure 1), and thus confound comparisons with earlier studies.

### SIF RCT Literature Review 1985–1994

Next, we searched PubMed with the keywords “Soy” AND “Infant” AND “Formula” and the filters “From 1985/01/01 to 1994/12/31” AND “Clinical Trial” and retrieved 52 citations (Table 2). This search was performed to enable comparison of cohorts consuming SIF before and after 1994 when genetically modified (GM) soy was introduced into the United States food supply. Six relevant trials published between 1985 and 1994 are now reviewed.

**Table 2:** **PubMed literature search with keywords “Soy” AND “Infant” AND “Formula” and filters “From 1985/01/01 to 1994/12/31” AND “Clinical Trial**.”

1.	Iron bioavailability studied in infants: the influence of phytic acid and ascorbic acid in infant formulas based on soy isolate	Davidsson et al. (30)
2.	Essential fatty acid metabolism and requirements for LBW infants	Uauy-Dagach et al. (31)
3.	Comparison of a rice-based, mixed diet versus a lactose-free, soy protein isolate formula in young children with acute diarrhea	Maulen-Radovan et al. (32)
4.	Nutritional evaluation of various protein hydrolyzate formulas in term infants during the first month of life	Rigo et al. (33)
5.	Evaluation of a maize-cowpea-palm oil diet for the dietary management of Nigerian children with acute, watery diarrhea	Grange et al. (34)
6.	Antigen-reduced infant formulas versus human milk: growth and metabolic parameters in the first 6 months of life	Giovannini et al. (35)
7.	Growth and protein status of term infants fed soy protein formulas differing in protein content	Churella et al. (36)
8.	Effect of dietary fat on cardiovascular risk factors in infancy	Fuchs et al. (37)
9.	Does early diet have an effect on subsequent macromolecular absorption and serum immunoglobulin E?	Juvonen et al. (38)
10.	Safety and efficacy of omega-3 fatty acids in the nutrition of very-low-birth-weight infants: soy oil and marine oil supplementation of formula	Uauy et al. (39)
11.	Dietary modifications versus dicyclomine hydrochloride in the treatment of severe infantile colics	Oggero et al. (40)
12.	Cow’s milk versus soy-based formula in mild and moderate diarrhea: a randomized, controlled trial	Allen et al. (41)
13.	Effects of infant nutrition on cholesterol synthesis rates	Cruz et al. (42)
14.	Nutrient absorption and weight gain in persistent diarrhea: comparison of a traditional rice-lentil/yogurt/milk diet with soy formula	Bhutta et al. (43)
15.	Influence of dietary manipulation on incidence of atopic disease in infants at risk	Bardare et al. (44)
16.	Effect of dietary fiber (soy polysaccharide) on the severity, duration, and nutritional outcome of acute, watery diarrhea in children	Brown et al. (45)
17.	Clinical evaluation of the tolerance for a soy-based special milk formula in children with cow’s milk protein intolerance/allergy	Buts et al. (46)
18.	Bone mineralization in the first year of life in infants fed human milk, cow milk formula, or soy-based formula	Mimouni et al. (47)
19.	Administration of rhesus-human reassortment tetravalent rotavirus vaccine in breast-fed infants	Ceyhan et al. (48)
20.	Docosahexaenoic acid status of term infants fed breast milk or infant formula containing soy oil or corn oil	Ponder et al. (49)
21.	Long-term prevention of allergic diseases by using protein hydrolyzate formula in at-risk infants	Mallet and Henocq (50)
22.	Bone mineral metabolism in full-term infants fed human milk, cow milk-based, and soy-based formulas	Venkataraman et al. (51)
23.	Gastric emptying using three different formulas in infants with gastroesophageal reflux	Tolia et al. (52)
24.	Dietary essential fatty acid supply and visual acuity development	Birch et al. (53)
25.	Nutritional management of persistent diarrhea: factors predicting clinical outcome	Bhutta et al. (54)
26.	Retinal development in very-low-birth-weight infants fed diets differing in omega-3 fatty acids	Birch et al. (55)
27.	Modulation of infant formula fat profile alters the low-density lipoprotein/high-density lipoprotein ratio and plasma fatty acid distribution relative to those with breast-feeding	Hayes et al. (56)
28.	Tolerance of a soy formula by infants and children	Nadasdi (57)
29.	Dietary management of persistent diarrhea: comparison of a traditional rice-lentil based diet with soy formula	Bhutta et al. (58)
30.	Evaluation of the effect of a fiber-enriched formula on infant colic	Treem et al. (59)
31.	Cumulative incidence of atopic disorders in high-risk infants fed whey hydrolyzate, soy, and conventional cow milk formulas	Chandra and Hamed (60)
32.	Role of a soy-based lactose-free formula in the outpatient management of diarrhea	Santosham et al. (61)
33.	Safety of casein hydrolyzate formula in children with cow milk allergy	Sampson et al. (62)
34.	Evaluation of a new Israel infant soy formula	Weizman (63)
35.	Clinical trial of home available, mixed diets versus a lactose-free, soy protein formula for the dietary management of acute childhood diarrhea	Alarcon et al. (64)
36.	Role of carnitine in utilization of dietary medium-chain triglycerides by term infants	Rebouche et al. (65)
37.	Absorption and retention in acute diarrhea	Mann et al. (66)
38.	Enteral nutrition in infants with congenital heart disease and growth failure	Schwarz et al. (67)
39.	A comparison of rice-based oral rehydration solution and “early feeding” for the treatment of acute diarrhea in infants	Santosham et al. (68)
40.	Effects of unrestricted diet on mild infantile diarrhea. A practice-based study	Margolis et al. (69)
41.	Colic and the effect of changing formulas: a double-blind, multiple-crossover study	Forsyth (70)
42.	Response to RIT 4,237 oral rotavirus vaccine in human milk, adapted- and soy formula-fed infants	Zoppi et al. (71)
43.	Oral refeeding following acute gastroenteritis: a clinical trial using four refeeding regimes	Quak et al. (72)
44.	Effect of feeding whey hydrolyzate, soy, and conventional cow milk formulas on incidence of atopic disease in high-risk infants	Chandra et al. (73)
45.	Influence of maternal diet during lactation and use of formula feeds on development of atopic eczema in high-risk infants	Chandra et al. (73)
46.	Low carnitine intake and altered lipid metabolism in infants	Olson et al. (74)
47.	Parental counseling compared with elimination of cow’s milk or soy milk protein for the treatment of infant colic syndrome: a randomized trial	Taubman (75)
48.	The effect of feeding four different formulas on stool weights in prolonged dehydrating infantile gastroenteritis	Rajah et al. (76)
49.	Chronic diarrhea and soy formulas. Inhibition of diarrhea by lactose	Donovan and Torres-Pinedo (77)
50.	Bone mineralization and growth in term infants fed soy-based or cow milk-based formula	Steichen and Tsang (78)
51.	Role of soy-based, lactose-free formula during treatment of acute diarrhea	Santosham et al. (79)
52.	Protein quality and quantity in preterm infants receiving the same energy intake	Darling et al. (80)

The Rigo study (33) was a prospective evaluation of the effects of protein hydrolyzate formulas, both whey and soy, on infant growth metrics during the first month of life. The population included 97 healthy infants who were enrolled shortly after birth. The study population was divided into six groups of newborn infants fed either human milk (*n* = 23), three different whey hydrolyzate formulas (#1, *n* = 13; #2, *n* = 10; #3, *n* = 13), soy-collagen hydrolyzate formula (*n* = 18), or whey–casein hydrolyzate formula (*n* = 20). If the mothers indicated an intent to breast-feed, infants were assigned to the human milk group. The remaining infants were assigned to a hydrolyzate formula groups. Anthropometric data were collected at birth and at the end of the first month of life. The independent variable was human milk or type of formula, and the dependent variables were growth parameters, biochemical indices of protein metabolism, and plasma amino acid concentrations. The outcome was soy-collagen hydrolyzate formula, which was associated with a significant decrease in weight and length as well as total serum protein and transferrin levels compared to the human milk cohort. Immunoglobulin G levels were significantly increased compared to the breast-fed cohort. Soy-collagen hydrolyzate formula led to an increase in several amino acids including glycine, hydroxyproline, citrulline, ornithine, arginine, aspartic acid, serine, threonine, and histidine as well as a decrease in lysine and cystine. Whey–casein hydrolyzate formula induced a plasma amino acid pattern closer to the human milk profile. Of note, the use of soy-collagen hydrolyzate formula makes it difficult to ascertain the effects of soy compared to the collagen.

The Giovannini study (35) was a prospective evaluation of growth and metabolic parameters in infants during the first 6 months of life in response to formula type. The study population included 82 infants with a family history of IgE-mediated disease. Infants were divided according to the type of milk feeding from the end of the first month of life up to 6 months: human milk (*n* = 29), SIF (*n* = 15), whey-based low-degree hydrolyzate (*n* = 15), casein-based high-degree hydrolyzate (*n* = 13), and soy plus collagen-based high-degree hydrolyzate (*n* = 10). At 0, 3, and 6 months of life, anthropometric measurements were taken. A fasting blood sample was obtained at 5 months of age before beginning introduction of solid foods. The independent variable was human milk or type of formula, and the dependent variables were anthropometric measures (weight, length, and head circumference) and biochemical parameters (serum hemoglobin, blood urea nitrogen, glucose, calcium, phosphorus, alkaline phosphatase, and transferrin). The outcome was a lower body mass index and higher blood urea nitrogen in the casein-based high-degree hydrolyzate group at 3 months and higher levels of essential amino acids in soy formula, whey-based low-degree hydrolyzate, and casein-based high-degree hydrolyzate groups. There were lower levels of branched-chain amino acids in all formula-fed infants compared to breast-fed.

The Churella study (36) was a blind, parallel RCT that measured growth and protein status in full-term infants fed SIF with varying protein concentration. The Abbott Laboratories-sponsored study enrolled 84 infants at 2 days of age who were free of genetic abnormalities and had no signs of cardiac, respiratory, gastrointestinal, or metabolic disease. Infants were randomly assigned formula that was the single food source until 112 days of age in a blind parallel study. Subjects, *n* = 20, were removed during the study for suspected formula intolerance, illness not related to the formula, poor growth due to gastroesophageal reflux, parents did not return infant for clinic visits, parents removed infant from study, or infant received other than the assigned feeding. The final analysis included 32 male and 32 female infants. The independent variable was type of formula (normal or lower soy protein), and the dependent variables were anthropometric (weight, length, and head circumference), plasma biomarkers (plasma urea nitrogen, total protein albumin, and transthyretin), and stool characteristics. The outcome was no statistically significant differences in weight, length, or head circumference. Plasma urea nitrogen levels were lower in infants fed the lower protein SIF. The other plasma measurements, tolerance to the two formulas and stool characteristics, were similar. Unfortunately, there was no non-soy control group for comparison. The widely used Enfamil ProSobee contains 2.5 g soy protein per 100 kcal of formula (Figure S1 in Supplementary Material), which is similar to the protein content of the lower soy protein formula employed in this study (2.45 g protein per 100 kcal).

The Mimouni study (47) was a single-blind, prospective trial evaluating bone mineralization in healthy infants fed human milk versus cow milk formula (Similac) or SIF (Isomil or ProSobee). All infants were selected from a population in which the mothers elected not to breast-feed. Breast-fed infants were recruited concurrently for comparison. Parents were aware of the formula assignments, but investigators taking the measurements were blind. Infants (*n* = 107) were enrolled with 59 completing the study through week 52 and 72 completing the study through week 16. The 72 infants completing the study through week 16 were included in the final analysis and consisted of breast-fed (*n* = 10), Similac (*n* = 20), Isomil (*n* = 21), and ProSobee (*n* = 21). The independent variable was formula type, and the dependent variables were anthropometrics, BMC, and plasma biomarkers. Gain in length was significantly greater in all formula groups compared to breast-fed infants through week 26. The difference was no longer significant at 1 year of age. BMC and bone width of the radius was similar among all groups. There were no differences in calcium, magnesium, or alkaline phosphatase among groups. Serum P levels were higher at the 8-week follow-up in formula fed versus breast-fed. The ProSobee group had higher serum 1,25-dihydroxyvitamin D at 8, 16, and 26 weeks.

The Venkataraman (51) study was a prospective assessment of bone mineral metabolism in full-term infants fed human milk versus cow milk-based or SIF. The study population included 56 healthy, full-term infants who were assigned to formula groups by a random block design to achieve equal distribution of genders between groups: breast milk (*n* = 17), cow milk-based formula (Similac, *n* = 19), and SIF (Isomil, *n* = 20). Infants were exclusively fed breast milk or formula fed for first 4 months. The independent variable was type of milk fed, and the dependent variables were anthropometric measurements, plasma biomarkers and BMC at 8, 16, and 24–26 weeks of age. The outcome was growth and most plasma biomarkers in the infants did not differ significantly among groups. Serum phosphorus was significantly lower in the breast-fed group at 8 weeks of age compared to the cow milk formula cohort. The BMC at 16 and 24–26 weeks was higher in the soy cohort than the breast-fed, and bone width was higher at 16 weeks in the soy group.

The Steichen study (78) was a prospective evaluation of bone mineralization and growth in full-term infants fed SIF or cow milk-based formula in the first year of life. Infants (*n* = 36) were enrolled and all but one completed the study. Subjects were randomly assigned to feeding groups (Isomil with iron, SIF) versus Similac with iron (cow milk-based) and received formula within 24 h of birth. Nutrition for the first 6 months of life was exclusively formula. The independent variable was formula type, and the dependent variables were anthropometrics, BMC, and bone widths of the radius and ulna. The outcome was no differences in weight, length, and head circumference among groups. The soy group had lower BMC at 3, 6, 9, and 12 months of age but values were similar to those in previously studied breast-fed infants with vitamin D supplementation.

Of note, growth metrics are the most widely used indicator of infant health. The few RCT that have examined the effects of SIF on growth metrics have been in healthy, full-term infants and differences have not been observed. There were two published studies that included anthropometric data on height, weight, and head circumference in response to breast milk versus cow milk based or SIF that performed measurements at similar ages. Both studies were conducted in the United States. One study was published in 1992 and the other in 2012 allowing for the comparison of Generation Y (the millennial generation) versus Generation Z (the digital generation) at 6 months of age. We compared their results to determine if there was any change in infant anthropometric measurements between generations in response to consumption of SIF pre- and post-1994 when GM soybeans were introduced into farming. There appear to be no statistically significant changes in height, weight, or head circumference at birth or after 6 months with respect to diet (Table 3). The Venkataraman study enrolled full-term, healthy infants delivered at the Oklahoma Memorial Hospital in Oklahoma City between October 1986 and August 1990 (51). The Andres study enrolled participants from the Beginnings Study between 2002 and 2010 at the Arkansas Children’s Nutrition Center (7). In both studies, complementary foods were not introduced before 4 months of age.

**Table 3:** **Comparison of infant growth metrics in response to soy-based infant formula, Generation Y versus Generation Z**.

Study	Generation Y (51)			Generation Z (7)		
Diet base	Breast*n* = 17	Cow*n* = 19	Soy*n* = 20	Breast*n* = 131	Cow *n* = 131	Soy*n* = 129
**At birth**						
Weight (kg)	3.37 ± 0.0	3.27 ± 0.09	3.35 ± 0.07	3.58 ± 0.34	3.51 ± 0.39	3.45 ± 0.37
Height (cm)	51 ± 0.6	50.8 ± 0.5	51.2 ± 0.6	51.6 ± 2.2	51.3 ± 2.5	51.2 ± 2.2
Head circumference (cm)	34.9 ± 0.3	34.2 ± 0.3	34.4 ± 0.4	ND	ND	ND
**At 6 months**						
Weight (kg)	7.4 ± 0.2	7.5 ± 0.2	7.9 ± 0.3	7.7 ± 0.9	8.0 ± 0.8	7.9 ± 0.8
Height (cm)	66.9 ± 0.9	66.6 ± 0.7	67.2 ± 1.0	66.2 ± 2.4	66.7 ± 2.3	66.6 ± 2.2
Head circumference (cm)	43.9 ± 0.3	43.7 ± 0.3	43.4 ± 0.5	43.5 ± 1.3	43.6 ± 0.2	43.8 ± 1.3

### SIF Systematic and Meta-Analysis Literature Review

In addition to the reviewed RCT, there has been one systematic review with meta-analysis entitled, “Safety of soya-based infant formulas in children” (81). The authors reviewed the safety of SIF in relation to anthropometric growth, BMC, immunity, cognition, and reproduction and endocrine function. The search strategy identified 156 potential studies published between 1909 and 2013 with 121 articles eliminated because they either covered non-related topics, were reviews, or did not contain sufficient extractable data. Studies were grouped based on pre- (11 studies published between 1960 and 1987) and post-fortification (11 studies published between 1992 and 2013) of SIF. The authors concluded, “Modern soy-based infant formulas are evidence-based safety options to feed children requiring them. The patterns of growth, bone health and metabolic, reproductive, endocrine, immune and neurological functions are similar to those observed in children fed cow milk formula or human milk.” Another extensive review of the SIF literature was conducted by an expert panel recruited by the National Toxicology Program Center for the Evaluation of Risks to Human Reproduction, which reviewed 28 published human studies and more than 120 laboratory animal studies that were considered to have utility for the safety evaluation of SIF (82). Overall, the panel recommended there was, “*minimal concern* for adverse effects on development in infants who consume soy infant formula.”

### Soy-Associated Health Benefits

The second argument in favor of developing therapeutic SIF is that soy protein is associated with health benefits in adults. The United States Food and Drug Administration (FDA) allows products with at least 6.25 g of soy protein to carry the health claim that they, “may help to lower the risk of cardiovascular disease.” The strength of the evidence to support this claim comes from an Evidence Analysis Library review in March of 2004 of studies regarding the effect of soy protein (26–50 g/day) on cholesterol levels in subjects with normal and elevated cholesterol (total cholesterol >200 mg/dL) and in subjects with diabetes. The review found that total serum cholesterol was reduced 0–20%, triglycerides were reduced 0–22%, and low-density lipoprotein (LDL) was reduced 4–24%. The overall strength of the evidence was given a Grade II (fair) and based on one high-quality meta-analysis plus two high-quality and two neutral-quality RCT that reported lower total cholesterol or LDL or both in adults who consumed at least 26 g of soy protein per day. There was one neutral-quality RCT that showed reduced high-density lipoprotein (HDL). Non-supporting studies included one high-quality meta-analysis and one high-quality RCT that found no soy-dependent differences in total cholesterol or LDL. The supporting high-quality meta-analysis included 38 controlled clinical trials on the effects of soy protein intake on serum lipids and indicated that the consumption of soy protein (average intake 47 g/day) instead of animal protein was associated with a decrease of 23.2 mg/dL (9.3%) in total cholesterol, 21.7 mg/dL (12.9%) in LDL, and 13.3 mg/dL (10.5%) in triglycerides (83). The Evidence Analysis Library also found that the effect of soy protein and/or isoflavones on total cholesterol, LDL, and HDL may vary based on baseline cholesterol levels and that there is no dose–response relationship between isoflavone consumption levels and effects on cholesterol. The Evidence Analysis Library recommends, “If consistent with patient preference and not contraindicated by risk/harms, then soy (e.g., isolated soy protein, textured soy, tofu) may be included as part of a cardioprotective diet. Consuming 26–50 g of soy protein per day in place of animal protein can reduce total cholesterol by 0–20% and LDL by 4–24%. Evidence is insufficient to establish a beneficial role of isoflavones as an independent component.”

In addition to reviewing available data on cholesterol effects, the Evidence Analysis Library reviewed nine RCT on the effect of soy (18–40 g/day) or isoflavone (70–143 mg/day) intake on blood pressure and found no effect in four trials, an increase in systolic blood pressure in women in one trial, and a decrease in systolic (4.3–18.4 mmHg) and diastolic (2.8–15.9 mmHg) blood pressure in four trials. They advise that, “the consumption of soy foods may or may not be beneficial for the reduction of blood pressure, since the effect of increased soy food intake on blood pressure is unclear.” They have determined that up to 30 g/day of soy protein consumed as supplements in the diet is well tolerated. Caveats are that soy may be an allergen, may not be recommended in those with or at high risk for breast cancer, and levels greater than 50 g/day may cause gastrointestinal distress.

Subsequent to the Evidence Analysis Library study, a meta-analysis found improved LDL, HDL, and fasting triacylglycerol levels suggesting that daily consumption of 15–30 g of soy significantly improved serum risk factors for cardiovascular disease (84). A meta-analysis of soy product consumption in patients with type 2 diabetes mellitus found that soy protein intake was beneficial in diabetic patients in terms of serum lipids, but there were no significant effects on fasting glucose, insulin, or glycated hemoglobin levels (85). In addition, consumption of soy or soy isoflavones is associated with reduced prostate cancer (86–89), gynecological cancers (90), and possibly breast cancer (91–96). Soy isoflavones stimulate bone formation, inhibit bone resorption, and increase bone mineral density resulting in attenuation of bone loss in menopausal women (97–101), albeit there are reports of only slight or no clinical effects (102).

### Biological and Economical Advantages of a SIF Drug Delivery Platform

The third argument in support of therapeutic SIF is that soybeans are an economical means to generate and deliver therapeutics. According to the American Soybean Association, soybeans were the second largest cash crop in the United States in 2012. Soybeans can be transfected with therapeutic genes of interest by biolistic gene delivery and then the soybeans produce the coded for proteins, which can be delivered to patients in a soymilk platform. Soy is the only plant protein source containing a complete amino acid profile. To determine if there was an association between the introduction of GM soybeans and the macro- and micronutrient content of soy protein, we analyzed data available from the United States Department of Agriculture National Nutrient Database for Standard Reference,^1^^,^^2^ which is the major source for food composition data in the United States. Release 26 (November, 2013) contains nutrient data for 8,463 food items and up to 150 food components. An analysis of the nutrient composition of soy protein isolate, the protein source in SIF, indicates that the proximate, mineral, vitamin, and lipid content of soy protein isolate have not changed from 1986 to 2013 (Table 4) GM soy was introduced into the food supply in the United States in 1995 and has currently grown to greater than 90% of soy production. Although not a controlled study, the non-variability in macro- and micronutrient content in soy protein isolate between 1986 and 2013 suggests that the genetic modification of soybeans has not altered these parameters.

**Table 4:** **Macro- and micronutrient content of soy protein isolate (1986–2013)**.

Proximates	Level (per 100 g)
Water (g)	4.98
Energy (kcal)	338
Protein (g)	80.69
Total lipid (g)	3.39
Carbohydrate (g)	7.36
Fiber (total dietary)^a^ (g)	5.6
Total sugars^b^ (g)	0
Ash (g)	3.58

**Minerals**	**Level (per 100 g)**

Calcium (mg)	178
Iron (mg)	14.5
Magnesium (mg)	39
Phosphorus (mg)	776
Potassium (mg)	81
Sodium (mg)	1,005
Zinc (mg)	4.03
Copper (mg)	1.599
Manganese (mg)	1.493
Selenium^c^ (mg)	0.8

**Vitamins**	**Level (per 100 g)**

Vitamin C (mg)	0
Thiamin (mg)	0.176
Riboflavin (mg)	0.1
Niacin (mg)	1.438
Vitamin B5 (mg)	0.06
Vitamin B6^a^ (mg)	0.1
Folate (mg)	176
Vitamin B12 (mg)	0
Vitamin A (IU)	0
Vitamin E^d^ (mg)	0
Vitamin D^e^ (IU)	0
Vitamin K^b^ (mg)	0
Choline (total)^f^ (mg)	190.9

**Lipids**	**Level (per 100 g)**

Fatty acids (total saturated) (g)	0.422
Fatty acids (total monounsaturated) (g)	0.645
Fatty acids (total polyunsaturated) (g)	1.648
Cholesterol (mg)	0

*Data available from ^a^1997–2013, ^b^2004–2013, ^c^1998–2013, ^d^2000–2013, ^e^2009–2013, ^f^2008–2013*.

## The Case Against Soy-Based Therapeutic Infant Formulas

The major argument opposing the use of SIF and therapeutic SIF is that safety has not been rigorously tested, particularly potential health effects associated with phytoestrogens, agrochemicals, and GM components. While the expert panel for the National Toxicology Program Center for the Evaluation of Risks to Human Reproduction concluded that there was minimal concern for adverse effects on development in infants consuming SIF, the decision was not unanimous (82). There was a dissenting opinion that: “(1) There are sufficient signals of adverse reproductive and developmental effects in experimental animals to worry about long-term effects on development of infants; (2) infants are exquisitely sensitive to the effects of exogenous chemicals during early life, and naturally occurring genistein in SIF has a much stronger estrogenic effect than non-natural estrogenic compounds on the developing reproductive system; and (3) the absence of evidence of an effect in human studies is not the same as evidence of absence of an effect, particularly given the paucity of human data to inform the conclusion.” Of the recent SIF RCT reviewed above, only four studies were longitudinal and only one assessed cognition. None of the studies assessed neurological outcomes or potential effects of agrochemicals, nor did they compare SIF versus GM SIF. All of the studies were conducted in healthy full-term infants, but infants genetically predisposed to developmental disabilities may be more susceptible to adverse chemical effects.

In general, the literature contains many conflicting reports regarding the health benefits of consuming soy and phytoestrogen supplements. The FDA recommends that 25 g per day of soy protein, as part of a diet low in saturated fat and cholesterol, may reduce the risk of heart disease. However, a review of 22 RCT comparing soy protein isolate to milk and other proteins on LDL cholesterol levels by the Nutrition Committee of the American Heart Association found that the average effect was only 3% (103). There was no benefit regarding HDL, triglycerides, or blood pressure. There is no conclusive evidence that soy phytoestrogens reduce hot flashes associated with menopause (91–96). In addition to the RCT and Evidence Analysis Library literature, a database search of the Cochrane Library with the keyword “Soy” retrieved 10 reviews and one protocol report, which are summarized in Table 5. Overall, these systematic and/or meta-analysis reports found no conclusive evidence supporting improved health with soy in terms of vasomotor effects in menopause, cholesterol levels in hypercholesterolemia, or allergies in infancy and childhood. There were limited data that genistein extracts may reduce hot flushes. There were no systematic reviews in the Cochrane Library regarding infant growth metrics in relation to soy. We did not observe a difference in growth metrics with SIF (Table 3), but there was only one 1992 and one 2012 study for comparison. A recent systematic review comparing milk-based formulas and SIF concluded that SIF are likely more carcinogenic; however, the evidence was limited (104). A study of isoflavone pharmacokinetics in rhesus monkeys indicated that metabolic and/or physiological immaturity in neonates reduces total clearance of soy isoflavones (105). Of note, although the FDA authorized a health claim linking the consumption of soy protein with reduced risk of coronary heart disease, the agency also lists soy in its Poisonous Plant Database with warnings regarding goiter, growth problems, amino acid deficiencies, mineral malabsorption, endocrine disruption, and carcinogenesis (106).

**Table 5:** **Summary of Cochrane Database Reports with keyword “Soy**.”

1.	Alternative lipid emulsions versus pure soy oil-based lipid emulsions for parenterally fed preterm infants (Review) P: Preterm infants (<37 weeks gestation) who received intravenous lipid emulsions as a part either of total parenteral nutrition or partial parenteral nutrition within the first week of life and for a minimum of 5 days I: S-LE C: Compare the effects of S-LE versus medium-chain triglyceride/long-chain triglycerides (MCT/LCT) LE, MCT-olive-fish-soy oil LE (MOFS-LE), MCT-fish-soy-oil LE (MFS-LE), olive-soy oil LE (OS-LE), borage-soy oil LE (BS-LE) on death, physical growth, chronic lung disease, and long-term neurodevelopmental outcomes O: There was a pooled effect toward decreased bronchopulmonary dysplasia (BPD) in OS-LE versus S-LE not reaching statistical significance. No difference in BPD was observed in any other comparison. There were no statistically significant differences in the primary outcomes of death, growth rate, or days to regain birth weight in any comparison	Kapoor et al. (107), Issue 12
2.	Lifestyle interventions for the treatment of urinary incontinence in adults (Review) P: Adults with urinary incontinence I: Weight loss, diet (soy-rich versus soy-free), fluid intake, reducing caffeine intake C: Compared lifestyle interventions for the treatment of urinary incontinence O: There is insufficient evidence to inform practice regarding the effectiveness of lifestyle interventions albeit available data show that evidence is growing for weight loss as a treatment to reduce urinary incontinence among morbidly and moderately obese women	Imamura et al. (108), Issue 12
3.	Dietary interventions (plant sterols, stanols, omega-3 fatty acids, soy protein, and dietary fibers) for familial hypercholesterolemia (Review) P: Children and adults with familial hypercholesterolemia I: Omega-3 fatty acids, soya proteins, plant sterols, or plant stanols C: Compared efficacy of cholesterol-lowering diet in reducing ischemic heart disease and lowering cholesterol with no dietary intervention and with supplements O: No conclusions could be made regarding effectiveness of a cholesterol-lowering diet or any other dietary interventions for familial hypercholesterolemia in regard to incidence of ischemic heart disease, number of deaths, and age at death	Malhotra et al. (109), Issue 6
4.	Phytoestrogens for menopause vasomotor systems (Review) P: Peri- and postmenopausal women without a history of breast cancer I: Soy phytoestrogens C: Compared the efficacy, safety, and acceptability of food products, extracts and dietary supplements containing high levels of phytoestrogens with no treatment, placebo, or hormone therapy O: There was a strong placebo effect, no conclusive evidence that phytoestrogen supplements were effective in reducing the severity or frequency of vasomoter symptoms associated with menopause, and four individual studies that could not be combined for meta-analysis but that suggested genistein extracts at greater than 30 mg/day reduce the frequency of hot flushes	Lethaby et al. (110), Issue 12
5.	Specially formulated foods for treating children with moderate acute malnutrition in low- and middle-income countries (Review) P: Children between 6 months and 5 years of age in low- and middle-income countries with MAM I: Specially formulated foods: LNS versus blended foods. LNS are food with high energy density and high lipid content. Blended foods are dry mixtures without high lipid content that can be cooked at home to make porridge or soup C: Compare the effectiveness and safety of specially formulated foods with standard care (medical care without foods) O: There was moderate to high-quality evidence that both LNS and blended foods are effective in treating MAM. There was no difference between LNS and blended foods in terms of number of deaths, progression to severe acute malnutrition, or drop out rate. LNS increased the number of recoveries and slightly improved nutritional status among those recovered Note: This study was retrieved during the search of the Cochrane database because soy was a component of the LNS and blended food	Lazzerini et al. (111), Issue 6
6.	Isoflavones for hypercholesterolemia in adults (Review) P: Hypercholesterolaemic postmenopausal women ranging in age from 40 to 83 years I: Isoflavones or SPI containing isoflavones C: Compared isoflavones versus placebo. Compared SPI plus isoflavones versus SPI alone O: There was no evidence that isoflavones affected total cholesterol, LDL, or HDL. There was a small statistically significant decrease in triglycerides with isoflavones alone but not with SPI	Qin et al. (112), Issue 6
7.	Polyunsaturated fatty acid (PUFA) supplementation in infancy for the prevention of allergy and food hypersensitivity (Protocol) P: Infants (cohorts fed: breast milk, cow milk formula, soy formula fed, hydrolyzed formula, complementary feeding) in their first year of life without clinical evidence of allergic disease at time of enrollment I: PUFA supplements C: Compare the effect of PUFAs in healthy, low risk, and high risk for allergy and food hypersensitivity infants. Compare the effect of PUFAs in low birth weight, preterm, and term infants. O: N/A: this was a Protocol study Note: Populations that have diets with elevated omega-3 PUFAs have a lower incidence of inflammatory conditions	Schindler et al. (113), Issue 9
8.	Probiotics for preventing acute URTI (Review) P: Infants, children, and adults around age 40 years I: Probiotics C: Compare the effectiveness and safety of probiotics with placebo in preventing acute URTI O: Meta-analysis indicated that probiotics were effective in reducing URTI and the number of associated antibiotic prescriptions, but there was no effect on the mean duration of an URTI episode Note: This study was retrieved during the search of the Cochrane database because probiotics are commonly consumed in fermented foods including yogurt and soy yogurt	Hao et al. (114), Issue 9
9.	Exercise for vasomotor menopausal symptoms (Review) P: Peri- and postmenopausal women I: Exercise C: Compare the effectiveness of any type of exercise intervention on the management of vasomotor menopausal symptoms O: In one small study that could not be included in the meta-analysis, hot flush scores were significantly lower in the exercise plus soy milk group than in the soy milk only group	Daley et al. (115), Issue 5
10.	Formulas containing hydrolyzed protein for prevention of allergy and food intolerance in infants (Review) P: Infants in the first 6 months of life without clinical evidence of allergy I: Hydrolyzed protein formula C: Compare the effect of feeding hydrolyzed protein formula to adapted cow milk or human breast milk on allergy and food intolerance O: There were no significant difference in infant allergy or childhood CMA and thus no evidence to support using hydrolyzed formula to prevent allergy compared to exclusive breast-feeding. With high-risk infants unable to breast feed, there was limited evidence that prolonged feeding with hydrolyzed formula may reduce infant and childhood allergy and infant CMA compared to cow milk formula	Osborn and Sinn (116), Issue 4
11.	Soy formula for prevention of allergy and food intolerance in infants (Review) P: Infants in the first 6 months of life without clinical evidence of allergy or food intolerance I: Adapted soy formula C: Compare the effect of feeding adapted soy formula to human milk, cow milk formula, or hydrolyzed protein formula in preventing allergy or food intolerance O: By meta-analysis, there were no significant difference in childhood allergy, asthma, eczema, or rhinitis	Osborn and Sinn (116), Issue 4

*S-LE, soybean oil-based lipid emulsions; MCT/LCT, medium-chain triglyceride/long-chain triglycerides LE; MOFS-LE, MCT-olive-fish-soy oil LE; MFS-LE, MCT-fish-soy-oil LE; OS-LE, olive-soy oil LE; BS-LE, borage-soy oil LE; BPD, bronchopulmonary dysplasia; MAM, moderate acute malnutrition; LNS, lipid-based nutrient supplements; SPI, soy protein isolate; PUFA, polyunsaturated fatty acid; URTI, upper respiratory tract infections*.

### Soy Contains Toxic Substances

Soy protein isolate comprises 14–16% of SIF by weight and contains many potentially toxic substances including phytoestrogens, saponins, protease inhibitors, and phytic acid that can interfere with digestion, reproduction, and thyroid function (82, 106, 117). Soy protein is rich is phytoestrogens including isoflavones such as genistein and daidzein. Genistein accounts for greater than 65% of the isoflavone content of SIF with total isoflavone concentrations ranging from 32 to 47 mg/L compared to human breast milk at 5.6 ± 4.4 mg/L (118). Infants fed SIF are exposed to 22–45 mg isoflavones per day (6–11 mg per kg body weight per day). High steady-state plasma concentration of isoflavones are maintained in infants by frequent feeding. These circulating isoflavone concentrations are 13,000–22,000-fold higher than plasma estradiol concentrations in early life. Chen and Rogan have estimated genistein consumption from SIF in terms of estrogenicity at about five contraceptive pills per day (119). The individual and total isoflavone content in SIF from Abbott, Enfamil, and PBM Products (powder form) were similar (Table 6). It should be noted, though, that nutrient databases are limited in their ability to estimate daily isoflavone intake as total isoflavone content varies significantly among soy protein isolates. Isoflavone content in soy products is highly variable due to differences in manufacturing processes (e.g., solvents, heating), analytical testing methods (e.g., solvents, enzymatic hydrolysis), and biological variability in the soybeans. Biological variability is the greatest variable and can be dependent on crop differences (e.g., growing conditions, maturity of soybeans, soybean strain, geographical location). Flavonoids and isoflavonoids occur in plants and food almost exclusively as glycosides, which are stable to most cooking methods, stomach acid, and secreted gastric enzymes (120). Deglycosylation can occur at several sites in the duodenum and jejunum and is a prerequisite for conjugation by intestinal enzymes and absorption by the small intestine. An analytical assessment of isoflavone content in soy milk and soy protein isolate over a 3-year period (1998–2000) indicated that isoflavone concentrations in soy protein isolate varied by 200–300%, but the protein content only varied by 3% (121). Isoflavone levels were not affected by storage of soy milk products for 359 days indicating that these bioactive compounds are highly stable; however, there is likely a two- to threefold variation in isoflavone content over a 6-month manufacturing period for any single branded soy product (121, 122). SIF in the United States have the highest phytoestrogen concentrations (genistein, daidzein, and glycitein) compared to the United Kingdom, Australia, New Zealand, and Brazil (82). Regarding the other potentially toxic chemicals in soy, saponins are soap-like foaming chemicals that are often bitter in taste. Heating SIF does not completely inactive the protease inhibitors. The phytic acid can bind to and prevent the absorption of minerals and niacin; hence, formulas must be fortified with iron, calcium, phosphorus, magnesium, zinc, manganese, copper, iodine, sodium, selenium, potassium chloride, choline, and inositol as well as vitamins A, C, D, E, K, and B (B1, B2, B6, B12, niacin, folic acid, pantothenic acid, and biotin). SIF contains high levels of manganese and aluminum (123–125).

**Table 6:** **Isoflavone content of soy protein isolate and powdered soy-based infant formulas**.

Nutrient	Mean^a^	*N*	SD	Min	Max
**Soy protein isolate**					
Daidzein	30.81	49	12.73	7.70	68.89
Genistein	57.28	55	14.17	27.17	105.10
Glycitein	8.54	42	3.22	5.4	26.40
Total isoflavones	91.05	49	26.00	46.5	199.25
**Infant formula (powder)**					
Abbott Similac with iron					
Daidzein	6.03	6	0.00	6.03	6.03
Genistein	12.23	6	0.51	11.43	13.03
Glycitein	2.73	6	0.02	2.70	2.77
Total isoflavones	25.82	11	2.85	20.17	31.60
Enfamil next step					
Daidzein	7.23	4	0.06	7.15	7.30
Genistein	14.75	4	0.20	14.50	15.00
Glycitein	3.00	4	0.04	2.95	3.05
Total isoflavones	25.00	4	0.08	24.90	25.10
PBM products bright beginnings					
Daidzein	5.70	2	ND	5.70	5.70
Genistein	13.55	2	ND	13.55	13.55
Glycitein	2.05	2	ND	2.05	2.05
Total isoflavones	28.01	7	2.95	21.30	30.70

*^a^mg/100 g*.

In addition to the potentially toxic substances found naturally in soy, soybeans undergo an extensive manufacturing process that can contaminate the soy protein with chemicals. An advertisement from Crown Iron Works Company that depicts the extensive chemical and heating steps involved in the soybean manufacturing process that produces soy protein isolate is reproduced with permission (Figure S2 in Supplementary Material). The process involves washing the soybeans with hexane followed by heat shock. The Total Diet Study, also known as the market basket study, is an ongoing FDA program that determines the level of various contaminants and nutrients in foods.^3^ Samples were collected between September 1991 and October 2003. Milk-based infant formula fortified with iron contains no detectable pesticide residues. Milk-based infant formula with low iron contains an average of 0.00052 ppm benzene, 0.00132 ppm chloroform, 0.00005 ppm styrene, and 0.00027 ppm toluene. SIF contains 0.00066 ppm benzene, 0.00120 ppm chloroform, 0.00001 ppm chlorpyrifos-methyl, 0.00005 ppm styrene, 0.00032 ppm toluene, and 0.00007 ppm xylene (*m*- and/or *p*-). The most recent market baskets collected between October 2003 and August 2005 did not include infant formula data. And unfortunately, the most commonly used pesticide, glyphosate, is not one of the contaminants tested by the Total Diet Study. Samsel and Seneff (126) found 170 ppb glyphosate in Enfamil ProSobee liquid concentrate.

Studies examining the health effects of soy are further complicated by the fact that 93% of soybean crops in the United States are GM (127). Glyphosate-tolerant soybeans were genetically engineered to express the 5-enolpyruvylshikimate-3-phosphate synthase gene from the CP4 strain of the bacteria *Agrobacterium tumefaciens*, which infers resistance to the Roundup^®^ herbicide glyphosate. Since the mid-1990s, there has been a steady increase in the use of GM soybeans in agriculture along with the concomitant use of glyphosate. Glyphosate is sprayed on GM crops where is acts as both a pesticide and herbicide as well as on other crops (for example, wheat) where it serves as a drying agent. This chemical accumulates in the soil, is not easily degraded, and is a known antibiotic and endocrine disruptor. Moms Across America found high levels of glyphosate in breast milk samples from lactating mothers.^4^ The increased use of glyphosate has been linked with numerous modern diseases (126, 128). There is a correlation coefficient of *R* = 0.99 when comparing the number of children with autism and the amount of glyphosate applied to corn and soy crops between the years 1991 and 2010 (126). It remains to be determined if bioactive components in soy have been altered by genetic modification. The United States Department of Agriculture supports the Database for the Isoflavone Content of Selected Foods. The latest version, Release 2.0, was released in September 2008 and provides daidzein, genistein, glycitein, and total isoflavone levels for soy protein isolate and SIF,^5^ which is summarized in Table 6. It is not possible to discern from these data if isoflavone levels have changed in response to genetic modification as Release 2.0 from 2008 is based on data from 1997, which is prior to widespread use of GM soy.

Glyphosate is absorbed by soybean plants with higher concentrations found in plants that are sprayed several times and when treatments occur during the flowering stage (129). Glyphosate can affect the nutritional content of soy as well as human immunoreactivity. Organic soybeans (glyphosate-free) exhibit higher sugar, total protein, and zinc levels and less fiber, total fat, and omega-6 fatty acids than GM soy (glyphosate-contaminated) (130). When sera from patients with a soy allergy was used to screen immunoblots of GM and wild soybeans, a unique 25 kDa protein band was observed with the GM soy (131). The USDA Pesticide Data Program is a national pesticide residue monitoring program that produces the most comprehensive pesticide residue database in the United States.^6^ The mission of the program is to help monitor the safety of America’s food supply. Between the years 1991 and 2015, glyphosate residue levels were only measured in one year (2011). Considering that glyphosate is the most commonly used pesticide in the United States, it is unfathomable and irresponsible that the USDA does not include this chemical in their yearly screening. In 2011, 300 soybean samples were tested and 271 (90.3%) contained glyphosate at levels ranging from 0.26 to 18.5 ppm.

### Soy Is Associated with Negative Health Outcomes

Diet has strong potential to affect health including neurological, immune, and endocrine function. Industrial soy foods have been implicated as a possible cause of Alzheimer’s disease with a log–log correlation of soy consumption and Alzheimer’s disease mortality of *r* = 0.24, *p* < 0.05 (132). It was long thought that large macromolecules do not pass directly from the digestive tract to the circulatory system. Emerging evidence is proving this paradigm false. Meal-derived DNA fragments that are large enough to carry intact genes can avoid degradation by the stomach acid, enter the human circulatory system, and are detected in blood plasma (133, 134). In addition to plant DNA, greater than 90% of soybeans are GM and carry bacterial genes that can code for toxic proteins. It has been estimated that if there was a one in a billion event of bacterial transformation of GM DNA into gut bacterium, that with 1E15 bacteria in the human gut, one would potentially have one million transformed bacteria in the intestines (127). These transformed bacteria could constitutively express toxic proteins that could damage the intestinal lining and cause disorders like celiac disease in which peptides enter the circulatory system, stimulate antibody production, and lead to autoimmune reactions that affect neurological function. The second most common GM trait codes for a built-in pesticide produced by the soil bacterium *Bacillus thuringiensis*.

There have been studies examining cognitive and reproductive development in infants fed SIF. Malloy and Berendes (135) tested 9- to 10-year-old children fed SIF or human milk during their first year of life and found no difference in intelligence quotient, behavioral problems, learning impairment, or emotional problems. A study by Strom and colleagues (136) surveyed adults aged 20–34 years old who had participated as infants in controlled feeding studies between 1965 and 1978 (Generation X). The study population included 811 subjects, which was 85% of the original cohort, including males (*n* = 120) and females (*n* = 128) who had been fed SIF. The outcomes were no correlation was found between infant formula use and education level, but women in the soy cohort reported longer duration of menstrual bleeding (about 8 h) and greater discomfort with menstruation. The soy cohorts also had a higher reported use of asthma or allergy drugs and greater tendency toward sedentary activities. Consumption of SIF has also been associated with breast development (137) and premature thelarche (133).

We found that soy-based diets are associated with increased propensity for seizures in both mouse and human models of neurological disease (138, 139). A literature search of the terms “soy” AND “seizure” produces very few studies. In humans, there are several case reports involving infants with seizure phenotypes in response to consumption of defective SIF or who developed micronutrient deficiencies due to SIF (reviewed in Data Sheet S2 in Supplementary Material). There have been several rodent studies assessing the effect of soy phytoestrogens on seizures. The problem with studying soy in rodents is that they metabolize phytoestrogens differently than humans. Thus, the data may not be generalizable between species. It should be noted that infants can efficiently digest, absorb, and excrete genistein and daidzein from SIF (140). Rodents conjugate isoflavones less efficiently and thus have higher circulating concentrations of biologically active forms (9). With this caveat noted, studies in rats and mice have demonstrated that soy increases seizures.

### Soy Diet Is Associated with Seizures in Rodents

Mohammadpour and colleagues (141) studied the effects of soy extract on pentylenetetrazole (PTZ)-induced seizures in the presence/absence of ovarian hormones in rats as well as gender-dependent differences in the effects of phytoestrogens on behavior. The study population included 72 Wistar rats, male and female, aged 2 months. The independent variable was soy extract, and the dependent variable was seizure latency. The cohorts included male/saline, male/low-dose soy, male/high-dose soy, sham/saline, sham/low-dose soy, sham/high-dose soy, ovariectomized/saline, ovariectomized/low-dose soy, and ovariectomized/high-dose soy. Low-dose soy groups were treated with 20 mg/kg soy extract for 2 weeks. High-dose soy groups were treated with 60 mg/kg soy extract for 2 weeks. Sham controls were injected with saline instead of soy extract. Seizures were induced with 90 mg/kg body weight PTZ (intraperitoneal injection). The latency to minimal clonic seizures and generalized tonic–clonic seizures were recorded. The outcome was that latency to minimal clonic seizure was shorter in male rats treated with either dose of soy extract and was dose dependent. Latency to generalized tonic–clonic seizure was shorter in male rats treated with either dose of soy extract, but not dose dependent. There was no effect with either dose of soy extract in female rats with either seizure type. The ovariectomized females responded similarly as males.

Work from our laboratory (138) compared seizure rates in several transgenic and knockout mouse models of neurological disease in response to a soy/grain-based versus a casein-based diet. We found significantly elevated seizure rates when the mice were fed soy-based diet. The study population was 452 C57BL/6 mice, male and female, aged postnatal day 21. The independent variables were diet (casein versus soy based) and genotype. The dependent variable was seizure propensity. The methodology included assessment of audiogenic seizure susceptibility by exposing mice to a 110 dB alarm for 5 min and measuring latency time to wild running, seizure, and death. The cohorts included wild-type, Tg2576, Ts65Dn, FRAXAD, and *Fmr1^KO^* mice. The outcome was that transgenic mouse lines that model fragile X syndrome, Alzheimer’s disease, and Down syndrome exhibit increased seizures when maintained on a soy-based compared to a casein-based diet. Supplementation of the casein diet with soy protein isolate or individual isoflavones did not statistically increase seizures in the Tg2576, although daidzein elicits a statistically significant increase in wild running in wild-type mice. Thus, a combination of dietary factors and/or agrochemicals may contribute to seizure susceptibility.

The Ebrahimzadeh Bideskan study (142) investigated the effects of soy extract on PTZ-induced seizures in ovariectomized rats. The study population included 60 Wistar female rats that were 2 months old. The rats were divided into four groups: sham, low-dose soy, high-dose soy, and ovariectomized. Each group was divided into two subgroups that received either a low dose of PTZ (40 mg/kg body weight, intraperitoneal) for 14 days or a single injection of PTZ (90 mg/kg body weight, intraperitoneal). The soy cohorts were injected with 20 and 60 mg/kg body weight of soy extract 30 min before each PTZ injection. The sham mice received saline instead of soy extract. The independent variable was soy extract, and the dependent variable was seizure propensity. The outcome was seizure scores, which were lower for the ovariectomized group than the sham group. Both of the soy groups had higher scores compared with the ovariectomized group. A single injection of high-dose PTZ significantly increased generalized tonic–clonic seizure but not minimal clonic seizure in the ovariectomized rats compared with sham. Treatment with either soy dose significantly decreased latency to generalized tonic–clonic seizure and minimal clonic seizure compared with ovariectomized group.

In addition to the aforementioned rodent studies, the effects of soy have been studied in monkeys and *in vitro*. Dietary soy is associated with epigenetic changes in monkeys such that overall methylation in liver and muscle tissue was increased when switching from a soy-based to casein-based diet (143). At high doses, genistein and daidzein are toxic to primary neuronal cultures (144).

### Soy Diet Is Associated with Seizures and Other Health Disorders in Humans

We conducted a retrospective analysis of the Simons Foundation Autism Research Initiative (SFARI) medical record database (139). There was a 2.6-fold higher rate of febrile seizures, a 2.1-fold higher rate of epilepsy comorbidity, and a 4-fold higher rate of simple partial seizures in autistic children fed SIF. In addition to seizures, there was an increased incidence of allergies, attention-deficit hyperactivity disorder, asthma, and bipolar disorder in the SIF cohort (Table 7). Limitations of the study included infant formula and disease data were based on parental recall, and there was a lack of data regarding potentially confounding issues such as why the subjects used SIF and for how long.

**Table 7:** **Increased incidence of comorbid disorders associated with the use of soy-based infant formula**.

	Soy		Non-soy			
	*N*	%	*N*	%	*P*^a^^,^^c^	CI^b^
**Females**						
Allergies	44	6.8	217	1.8	0.1	0.7–22
ADHD	44	6.8	217	2.8	0.2	0.5–12
Asthma	44	4.5	217	9.2	0.5	0.1–2.2
Bipolar	42	14.3	217	15.7	1.0	0.3–2.5
**Males**						
Allergies	297	3.0	1,391	1.4	0.06	0.9–5.0
ADHD	297	6.7	1,391	4.0	0.04	1.0–3.0
Asthma	297	8.4	1,391	4.9	0.02	1.1–2.9
Bipolar	273	23.8	1,390	15.2	0	1.3–2.4
**Genders combined**						
Allergies	341	3.5	1,608	1.5	0.01	1.1–5.2
ADHD	341	6.7	1,608	3.9	0.04	1.0–3.0
Asthma	341	7.9	1,608	5.5	0.08	0.9–2.4
Bipolar	315	22.5	1,607	15.2	0.001	1.2–2.2

*^a^Fisher’s exact test*.

*^b^95% confidence intervals*.

*^c^Chi-squared analysis*.

Maternal health, ethnicity, and geography likely play roles in the choice to feed SIF. Autoimmune disease in mothers with the fragile X mental retardation gene premutation is associated with seizures in their children with fragile X syndrome (145). In the SFARI autism population, seizure incidence was not more prevalent in the proband if the mother had an autoimmune disorder (Table S1 in Supplementary Material). However, autistic children reported to have been fed SIF were 3.5-fold (females) and 2.3-fold (males) more likely to have mothers with an autoimmune disorder (Table S2 in Supplementary Material). Thus, when the mother had an autoimmune disorder, her autistic child was twice as likely to have been fed SIF (31 versus 16%). The lack of an association between proband seizure incidence and maternal autoimmune status suggests that maternal autoimmunity is not a confounding factor for seizure prevalence in response to SIF. Although the number of subjects was small, there was a trend for increased use of SIF among African-Americans (Table S3 in Supplementary Material). There was a twofold increase in the percentage of children fed SIF in central North America (24%) versus both eastern (14%) and western (12%) North America (*P* = 0 for both comparisons). The percentage of autistic subjects from the SFARI database from central North America was 36.8%; yet, this population accounted for 51.2% of the subjects fed SIF suggesting that SIFs are disproportionately used in the central United States. Regarding SES factors in the SFARI population, the education of the mothers was not statistically different dependent on SIF use in their autistic children (66% of mothers of females fed non-soy-based formula graduated from college or higher, 61% for males fed non-soy-based formula, 56% for females fed SIF, and 58% for males fed SIF).

In addition to neurological and immune health, soy isoflavones affect development of the intestines as well as the makeup of the intestinal microbiota (146). There have been two studies investigating the influence of SIF on the gut microflora in infants and/or children. The first study found increased equol excretion in the soy group (19%) compared to controls (5%) with elevated *Bifidobacteria, Bacteroides*, and *Clostridia* bacteria in fecal samples from the soy group (147). The second study found elevated *Bifidobacteria* species (*B. adolescentis* and *B. infantis*), which were not detected before commencing the SIF (148). *Bifidobaterium, Bacteriodes*, and *Clostridium* are among the human intestinal bacteria that can produce *S*-equol (149, 150). *S*-equol is the biologically active metabolite of daidzein. Intestinal bacteria transform daidzein to equol in humans that are equol producers. In Japan, Korea, and China, up to 80% of people are equol producers, but as few as 25% of people in North America and Europe can biotransform daidzein into equol (150). Equol modulates expression of the BRCA1 and BRCA2 breast cancer genes through an epigenetic mechanism resulting in decreased methylation, which results in an increased level of oncosuppressors in breast cancer cell lines (151). The effect of equol on the methylation of neuronal genes has not been studied.

**Figure 2:**
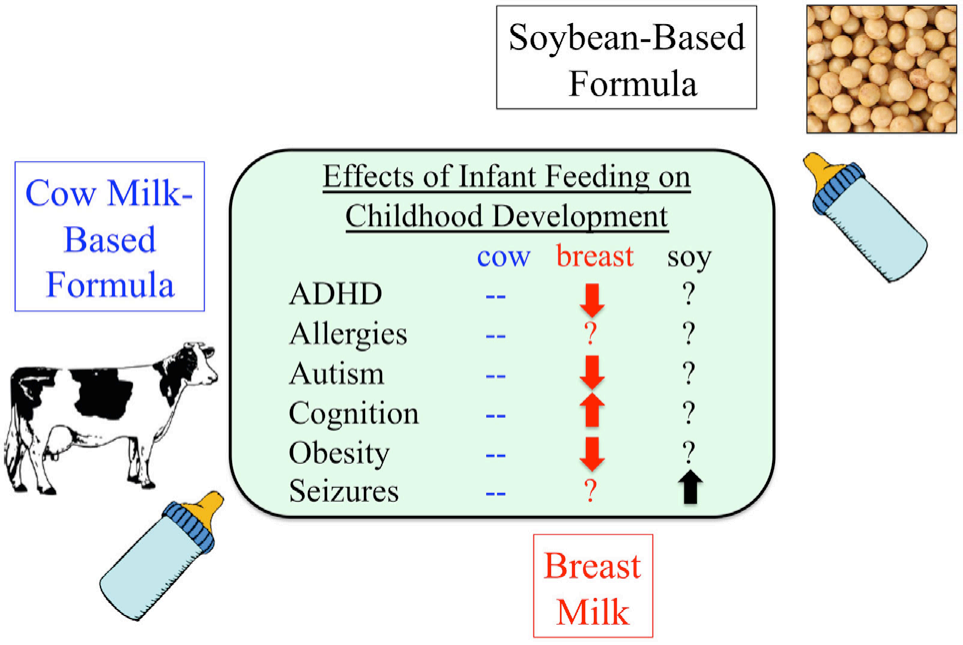
**Infant formula and disease**. As many as 25% of infants consume soy-based infant formula during their first year of life, but there is a paucity of information regarding potential adverse health effects (139, 152–158).

In summary, much remains to be learned regarding the effects of soy, soy constituents, and contaminating agrochemicals on health. There are conflicting reports in the literature regarding the health benefits associated with soy consumption in adults. There is a dearth of studies examining potentially toxic components and contaminants of soy. There is emerging data regarding adverse health effects associated with soy consumption. SIF RCT to date have involved healthy infants or infants with allergies, persistent diarrhea, or atopic dermatitis. There are no published trials assessing the effects of SIF on children with developmental disabilities. These vulnerable groups may be more susceptible to seizure promoting ingredients and/or chemicals in the diet. Overall, lack of knowledge related to the effects of SIF and agrochemicals on health could be equated with the state of affairs of cigarette smoking in the 1950s when smoking was generally regarded as safe. Over 50 years after the Surgeon General first warned of the health hazards of smoking, tobacco use is still a large burden in the United States where 18% of adults smoke.^7^ Decades later, there are no RCT that prove smoking causes lung cancer; however, enough evidence has accumulated where an RCT would be unethical. One could envision a similar scenario unfolding as evidence accumulates regarding adverse health effects associated with agrochemicals and GM food. A summary of the known effects of infant feeding on childhood development is provided in Figure 2.

## Transgenic EGF SIF to Treat Necrotizing Enterocolitis

*PLoS One* recently published an article by He and colleagues entitled, “Transgenic Soybean Production of Bioactive Human Epidermal Growth Factor (EGF),” in which the authors propose that soybean seeds could be utilized as a biofactory for the production of therapeutic agents such as epidermal growth factor (EGF), which could then be delivered to infants in a soymilk platform for the treatment of diseases such as necrotizing enterocolitis (NEC) (159). NEC, the second most common cause of death in premature infants, is a serious condition that occurs when the intestinal tissue is damaged and begins to die (160). The mission to prevent or treat devastating disorders including NEC is laudable. However, the strategy in this article raises several serious concerns.

First, the American Academy of Pediatrics concluded that SIFs are not recommended for preterm infants due to increased risk of osteopenia (161). SIFs are also not indicated in the management of documented cow milk protein-induced enteropathy or enterocolitis because of the high frequency of sensitivity to both cow milk and soy antigens in infants (161). Breast milk feedings are associated with a decrease in NEC in premature infants. Thus, a soymilk platform for drug delivery would not be recommended to prevent NEC.

Second, there are strong data that EGF is protective against the development of NEC; however, that evidence comes in the context of breast milk (162). Breast milk inhibits lipopolysaccharide receptor, toll-like receptor 4, within intestinal epithelium, but not in mice lacking epidermal growth factor receptor (EGFR). Overexpression of toll-like receptor 4 in the intestinal epithelium reversed the protective effects of breast milk. Selective removal of EGF from breast milk reduced the protective properties. To our knowledge, there ares no data examining the effects of EGF supplementation in a SIF platform on NEC or any other disorder. There is a wealth of literature with respect to the effects of isoflavones on EGFR signaling. Importantly, the most prevalent isoflavone in soy is genistein, which can reduce EGF-mediated DNA synthesis (163), decrease EGFR levels (164, 165), decrease or increase phosphorylation of EGFR (166, 167), and transactivate EGFR (168).

Third, there are several questionable data interpretation issues with this article. In their Figure 3, the authors claim that the human EGF produced in soybeans is the same molecular weight as commercially available recombinant human EGF; however, the recombinant EGF appears at least 0.5 kDa larger by immunoblot analysis (159). In their Figure 4, mass spectrometry analysis indicated that exact matches for peptides encompassing the majority (not all) of the complete mature soy EGF were obtained (159). The authors recognize that plant tissues possess biologically active components that could mask or enhance the effects of the soy EGF. In their Figure 5, they claim that SIF is biologically inactive because it does not modify the internalization, degradation, or phosphorylation of EGFR in HeLa cells (159). This finding is surprising considering the published literature regarding the effects of genistein on EGFR and suggests that HeLa cells are not the appropriate cell type to discern SIF-induced biological activities. In their Figure 6, the authors claim that there are insignificant differences in the small molecule profile between non-transgenic soybeans and those producing EGF except for the metabolites involving sulfur amino acid metabolism. There is a 19.4-fold increase in *S*-methylmethionine, an 8.24-fold increase in glucorante, and a 1.65-fold increase in skikimate (159). However, a closer analysis of their non-targeted metabolome set indicates that there are statistically significant increases in 10 of 20 amino acids (Table 8). Infants who have NEC have selective amino acid deficiencies, such as glutamine and arginine, that may predispose to the illness (169). All of the statistically significant differences between the EGF and non-transgenic soybeans were toward an increased level in the transgenic samples indicating that deficiencies in these amino acids should not be an issue; however, the effect of altered ratios of the amino acids is not known.

**Table 8:** **Secondary analysis of He et al. non-targeted metabolome set (159)**.

Fold change (epidermal growth factor/WT)	
**Charged amino acids**	
Arginine	0.71
Lysine	1.49*
Aspartic acid	1.25
Glutamic acid	1.19
**Polar amino acids**	
Glutamine	1.14
Asparagine	0.48
Histidine	1.41*
Serine	1.04
Threonine	1.19*
Tyrosine	0.82
Cysteine	1.2
Methionine	1.22*
Tryptophan	1.34*
**Hydrophobic amino acids**	
Alanine	1.26*
Isoleucine	1.55*
Leucine	1.09
Phenylalanine	1.08
Valine	1.38*
Proline	1.29*
Glycine	1.29*

*P < 0.05 by Welch’s two-sample t-Test*.

Additional concerns include the strong promoter and terminator used to regulate gene expression of EGF in soybeans as well as the hygromycin resistance gene in the construct. Meal-derived DNA fragments that are large enough to carry complete genes can avoid degradation in the digestive system and pass into human blood (134). Theoretically, the soy EGF construct could be transformed into gut bacteria where it could constitutively produce EGF (127). The odds of bacterial transformation in preterm infants may be greater due to an underdeveloped microbiota.

## Testable Hypotheses to Support/Negate the Development of Therapeutic SIF

Testable hypotheses to support the development of therapeutic SIF (*written in the alternative*): (1) *SIF causes altered reproductive, immune system, and/or cognitive development*. This hypothesis can be addressed through longitudinal and retrospective RCT. For example, The Beginnings cohort can be tested at adolescent and adult ages. The Andres study (2) did not find alterations in reproductive organs at 5 years of age in response to SIF; however, puberty may be a more appropriate age to discern effects. Soy-based diets can affect reproductive tissue growth and hormone secretion in male rats (170). In addition to reproductive organs, allergy and cognitive testing at multiple stages of development could discern differences in immune system and cognitive development. The retrospective study by Strom and colleagues (136) found associations with menstrual, asthma, and allergy phenotypes in relation to infant diet. Our retrospective analysis of the SFARI medical record database found a 3.5-point decrease in intelligence quotient in autistic girls fed SIF (not statistically significant in a cohort of 253 subjects). Rhesus monkeys fed SIF do not show effects on growth, health, developmental milestones, temperament ratings, or stereotypy but do exhibit altered diurnal rhythms and reduced play behavior (171). Secondary analyses of national survey data such as the National Health and Nutrition Examination Survey data, a nationally representative population of 1,864 infants in which 12% of babies consumed SIF, could identify associations between infant diet and comorbid disorders (172).

(2) *Glyphosate is associated with adverse health effects*. This hypothesis can be addressed through longitudinal RCT with subjects randomly assigned to organic versus conventional diets. Comorbid disorders of particular interest for the study are autism, diabetes, thyroid cancer, and Alzheimer’s and Parkinson’s disease where incidence or death rates have grown rapidly since the introduction of GM food and use of glyphosate (126). In addition to RCT, the potential long-term effects of glyphosate could be studied in animal models, particularly in regard to body composition, obesity, and micronutrient deficiencies/toxicity. For example, glyphosate disrupts cytochrome P450 enzymes and is associated with manganese deficiency and toxicity (126). SIFs are naturally high in manganese (up to 200× higher than breast milk), and while an essential nutrient, it can also act as neurotoxin (173, 174). The combination of SIF and glyphosate and their combined effects on manganese levels may exacerbate adverse health effects similar to the exponential increase in lung cancer with combined exposure to cigarette smoking and asbestos.

(3) *SIF and glyphosate adversely affect the human gut microbiota*, and (4) *GM DNA is transferred to human gut bacteria*. These hypotheses could be tested by screening the bacterial content of human fecal samples as a function of diet. There is evidence from women in Canada who ate a typical diet including GM soy, corn, and potatoes of the presence of the bacterial toxin *B. thuringiensis* in maternal and fetal blood (175). Thus, the toxin can cross the placenta. It remains to be determined if the pesticides in the blood came directly from the food and/or if GM DNA was incorporated into gut bacteria to form pesticide-producing factories in the intestines.

And (5) *adverse health effects associated with SIF and glyphosate are more detrimental in individuals genetically predisposed to developmental disabilities*. This hypothesis could be tested in transgenic animal models or by retrospective analysis of medical records. Porcine or primate models would be more advantageous than rodents, which conjugate isoflavones less efficiently.

## Public Policy Discussion

In 1826, Anthelme Brillat-Savarin said, “Tell me what you eat and I will tell you what you are.” The field of food science is rapidly evolving. As a society, we have moved in fast forward away from knowing what we are eating or where and how it is produced. Food replicators featured in the futuristic television shows *The Jetsons* and *Star Trek* are here in the form of 3D food printers. Refrigerators can identify spoiled food and order groceries. But unlike *Star Trek*, technology has not advanced to where we have medical tricorders to instantly diagnose the effects of environmental exposures including diet and agrochemicals on human health. Considering the importance of diet to child development, a public discussion on the merits of therapeutic SIF is in order. Issues that need to be addressed include the following: (1) rigorous and longitudinal study of the biological effects of endogenous and contaminating components of SIF and therapeutic SIF on health, (2) identification of disease populations that may benefit or be harmed from therapeutic SIF, and (3) the ethics of national supplementation of food products.

### Rigorous and Longitudinal Study

Rigorous and longitudinal study of the biological effects of soy, both conventional and GM, on development and disease propensity is required. The current literature consists of numerous conflicting reports and potentially confounding variables regarding soy consumption. Therapeutic SIF would presumably be considered a medical food product, which are consumed under medical supervision but do not require a prescription. We need to discuss as a society what level of evidence is required to prove safety and efficacy of these types of products, and then, we need to conduct studies at that level prior to public consumption. A large percentage of the population will not be satisfied with FDA oversight alone. At the national level, we are in the midst of a heated debate regarding GM foods, which have been approved by the FDA. The Mellman Group found that 88% of Americans are in favor of requiring labels for foods that contain GM ingredients. Sixty-four other countries have the right to know whether their food is GM. Yet in 2016, the United States Senate and House of Representatives voted in favor of the Safe and Accurate Food Labeling Act (a.k.a. the DARK Act, Deny Americans the Right-to-Know Act), which blocks states from issuing mandatory food labeling laws for GM foods. The effects of GM soy consumption have not been studied in humans and are not monitored. Widespread farming of GM crops has exponentially increased glyphosate usage (126), the effects of which have also not been studied nor monitored. An unanticipated problem of glyphosate usage on GM crops was that weeds growing among the crops became resistant to the herbicide (176), which has spurred increased application of the chemical over time for weed control.

### Identification of Vulnerable Disease Populations

We need to identify populations that may benefit or be harmed from therapeutic SIF. Our understanding at this time of how diet interacts with genes, gender, and ethnicity is very limited. Of interest, in the Andres study (2), the parents were allowed to choose the diets they were going to feed their infant before enrollment in the study. African-Americans constituted 8.9% of the total study population (9 of 101 subjects); yet, parents of eight of those subjects chose to feed their infants SIF resulting in a significant increase in African-American representation in the SIF cohort (23.5%) compared to 0% in the breast-fed and 3.1% in the cow milk-based cohort. Our analysis of the SFARI medical records also indicated that an increased percentage of African-American infants were fed SIF (139). This raises important concerns regarding SES factors that may affect formula choice and disease outcomes. Because of perceived lactose intolerance, African-American babies may be more likely to receive SIF. NEC is three-fold higher in African-Americans than Caucasians (160), and SIF is not recommended for treatment. African-American girls enter puberty and reach menarche earlier than Caucasian and Hispanic girls (177). SIF is associated with very early as well as late menarche (178, 179), and with heavy menstrual bleeding and larger uterine fibroids among African-American women (180, 181). The Andres study (2) tested reproductive organ size at 5 years of age with a planned follow-up study at puberty. The data collected in the follow-up study can be compared with any reported menstrual problems. Analysis of the National Health and Nutrition Examination Survey data indicated that higher consumption of SIF was associated with increased family income and level of education (172). SIF is widely used in Israel, where it constitutes 31.5% of infant formula sales (70% of children receive soy for greater than 6 months) (182). There is a high rate of ADHD (12.6%) in Israel (183). Thus, there may be ethnic and SES populations that are more likely to consume SIF and thus more susceptible to adverse health effects.

### The Ethics of National Supplementation of Food

Finally, the ethics of supplementation of food products needs to be discussed. Concerns include containment of GM crops and national fortification of foods for population-wide consumption. If therapeutic soybeans are grown in the open, cross pollination between neighboring farms could contaminant non-transgenic crops. If a micronutrient or protein of interest is deemed beneficial to health, it could be mass produced in soybeans. Soy protein is already extensively used as an additive by the food industry in the United States in nearly all types of foods. Two current examples of national supplementation of our food and water are folic acid and fluoride, and both are associated with adverse health effects in some people.

In 1998, the FDA began requiring food manufacturers to enrich grain products with folic acid with the goal of preventing neural tube birth defects. Folate is found naturally in leafy green vegetables and citrus fruits, but not all pregnant women were consuming enough resulting in birth defects such as spina bifida due to folate deficiency. Folic acid is an inactive, more stable form of folate that is added to food. Normally, the body converts folic acid to folate; however, persons with mutations in the methylenetetrahydrofolate reductase gene cannot process folic acid and it builds up in the body. In the United States, up to 60% of the population is heterozygous and up to 25% homozygous for methylenetetrahydrofolate reductase genetic mutations (184). Most individuals are unaware that they have these gene defects, and consumption of excess folic acid may increase the risk for miscarriages, autism in offspring^8^ (185), and some types of cancer (186, 187). In addition, folic acid can interact with medications such as antiepileptic drugs. Thus, while supplementation of the national food supply with folic acid has decreased neural tube defects by 25–30% (188), it may be causing health problems for other portions of the population. Similarly, fluoride supplementation has a modest benefit in reducing dental caries, but is associated with decreased intellectual ability, hypothyroidism, dental and skeletal fluorosis, enzyme and electrolyte derangement, and uterine cancer (189). There is a significant problem in obtaining the optimal dose of bioactive agents when using food or water supplementation because exposure is not controlled.

Overall in 2016, we are far from understanding the effects (good or bad) of food on health and disease and could be exacerbating disorders like autism, Alzheimer’s disease, and cancer by increased exposure to chemicals through modern food growth and manufacturing processes. A critical review of the marketing claims on infant formula products in the United States indicates that there is insufficient evidence to support claims regarding reducing lactose, using hydrolyzed or soy protein, or adding pre-/probiotics to formula to aid in reducing fussiness, gas, or colic (190). Therapeutic SIF may be technologically feasible and economical; however, are they safe and biologically relevant?

Both sides in this debate could argue that there is a lack of funding to conduct the rigorous studies required to prove their positions. A search of the National Institutes of Health Reporter website with the term “soy infant formula” indicates only five funded grants. Those applications are studying the effect of SIF on the prevention of uterine fibroids, establishment of the gut microbiota, and the growth and development of estrogen-responsive tissue. In addition, there are studies on the effect of neonatal genistein exposure on glucocorticoid receptor signaling and function in the uterus and how genistein affects female reproductive tract development and function. A search with the term “genetically modified soy” returns a study on the allergenicity of genetically modified food proteins, a study testing the effect of Roundup on neuronal degeneration in nematodes (worms), and a funded core facility to maintain a greenhouse for pharmaceutical producing plants. The remaining search results involved genetically modified animals and were not studying the effects of a genetically modified food product. A search with the term “glyphosate” returned two studies examining occupational exposure to pesticides and the risk of cancer. Thus, much remains unstudied regarding the health effects of SIF, associated agrochemicals, and therapeutic SIF.

In summary, SIF constitute approximately 12% of the infant formula market (82). Approximately a quarter of babies are fed SIF for some time during their first year of life, although data are lacking regarding how many are exclusively fed SIF (117). Proponents of therapeutic SIF argue that the long time and widespread use of SIF in conjunction with normal growth metrics suggest that these formulas are a safe, healthy, and inexpensive alternative to cow milk-based infant formula. Opponents believe more rigorous study is required. The question is do we chance error on the side of technology, economics, and innovation and move forward with the development of therapeutic SIF or do we take a more prudent approach and put proven safety and efficacy first.

## Ethics Statement

All mouse husbandry and euthanasia procedures were performed in accordance with NIH and an approved University of Wisconsin–Madison animal care protocol administered through the University of Wisconsin Research Animal Resources Center. The institutional review protocol governing the Simons Simplex Collection was approved by the Institutional Review Board at Columbia University Medical Center. Written informed consent was provided by all guardians or research subjects. The privacy of participants was protected by using global unique identifiers. The research protocol for using the Simons Simplex Collection in this study was approved by the Human Research Protection Program at the University of Wisconsin–Madison, which determined that the study qualified for exemption under category: 45 CFR 46.101(b)(4). Not applicable. Non-identified data from a medical record database were used by the author. The non-identified study population was autistic minors.

## Author Contributions

CW conceived, drafted, critically revised, and gave final approval for publication of the manuscript.

## Conflict of Interest Statement

The author declares that the research was conducted in the absence of any commercial or financial relationships that could be construed as a potential conflict of interest.

## Footnotes

^1^United States Department of Agriculture (2013) USDA National Nutrient Database for Standard Reference, release 26, http://www.ars.usda.gov/Services/docs.htm?docid=8964.

^2^United States Department of Agriculture (1986) USDA Agriculture Handbook.

^3^FDA market basket study, http://www.fda.gov/Food/FoodScienceResearch/TotalDietStudy/ucm184232.htm.

^4^Moms Across America, http://www.momsacrossamerica.com/tags/glyphosate.

^5^Bhagwat S (2008) USDA database for the isoflavone content of selected foods, release 2.0. Nutrient data laboratory home page, http://www.ars.usda.gov/nutrientdata/isoflav.

^6^USDA Pesticide Data Program, https://www.ams.usda.gov/datasets/pdp.

^7^The Health Consequences of Smoking – 50 years of Progress. A report of the Surgeon General, http://www.surgeongeneral.gov/library/reports/50-years-of-progress/exec-summary.pdf.

^8^Johns Hopkins University Bloomberg School of Public Health. Too much folate in pregnant women increases risk for autism, study suggests: Researchers say that while folate deficiency is bad for developing fetus, excessive amounts could also be harmful. ScienceDaily, www.sciencedaily.com/releases/2016/05/160511105352.htm.
