# Supplementary material for: Soy-Based Therapeutic Baby Formulas: Testable Hypotheses Regarding the Pros and Cons
Source: Front Nutr. 2017 Jan 18;3:59. doi: 10.3389/fnut.2016.00059 (PMC5241282; doi:10.3389/fnut.2016.00059)
Supplement: Supplementary file 1 [file Data_Sheet_1.DOCX]

Supplementary Material

**Soy-based therapeutic baby formulas: testable hypotheses regarding the pros and cons**

Cara J. Westmark*

*** Correspondence:** Cara J. Westmark: westmark@facstaff.wisc.edu

# Supplementary Text 1

The Lasekan study (Lasekan et al., 2015) was a randomized, double-blind, multi-center, 28 day parallel feeding trial conducted by Abbott Nutrition that compared gastrointestinal tolerance and hydration in healthy term newborn infants in response to supplementation of SIF with short-chain fructooligosaccharides. The population included 188 healthy term infants age 0-8 days of age randomized into 3 formula groups: [1] 62 infants in the control group were fed commercialized SIF containing sucrose as 20% of total carbohydrate [Similac® Isomil® Advance®], no supplemental short-chain fructooligosaccharides and no mixed carotenoids [lutein, lycopene, beta-carotene]; [2] 64 infants in an experimental group were fed SIF containing sucrose plus short-chain fructooligosaccharides [2.5 g/L] and mixed carotenoids; [3] and 62 infants in an experimental group were fed SIF containing short-chain fructooligosaccharides [2.5 g/L] and mixed carotenoids with no sucrose. Demographic data included gender and race but not SES or parental education. The independent variable was formula choice and the dependent variable was mean rank stool consistency. The outcome was no significant differences were found in growth [weight, length, head circumference], mean rank stool consistency, stool frequency, formula intake, spit-up/vomit, urine specific gravity, hydration status or adverse effects between groups.

The Skau study (Skau et al., 2014) was a cross-sectional survey infants to predict whether complementary food products can ensure dietary adequacy. The study population included 78 Cambodian infants [6-11 months old]. The Optifood software tool, which utilizes linear programming to formulate food-based recommendations, was used to model baseline diet and 4 formulated complementary food products [WinFood, WinFood-Lite, Corn-Soy-Blend Plus, and Corn-Soy-Blend Plus Plus], which were added to the diet at 33 g/day dry weight for infants aged 6-8 months and 40 g/day for infants aged 9-11 months. The WinFood diets are nutritionally improved food products for infants and young children based on locally available foods. The WinFood contains rice, dried small fish and edible spiders. The WindFood-Lite contains rice, dried small fish, and a mineral and vitamin premix. Dietary data were collected by 24 hour recall. Infants were stratified into 2 age groups [6-8 and 9-11 months] due to age-dependent energy differences. The independent variable was diet and the dependent variable was nutrient intake. The outcome was that the baseline diet did not achieve Recommended Nutrient Intake for thiamin, riboflavin, niacin, folate, vitamin B-12, calcium, iron, and zinc and none of the complimentary food products covered the gap for thiamin, niacin, iron and folate. Only the WinFood and Winfood-Lite filled the nutrient gap for calcium. The WinFood-Lite and both Corn-Soy-Blend diets filled the nutrient gap for zinc.

The Setchell study (Setchell et al., 2011) determined whether soy isoflavone metabolism differs between humans and rodents. Mass spectrometry was used to determine circulating total and unconjugated isoflavone concentrations in plasma in 7 cohorts including: [1] Sprague Dawley rats and 3 strains of mice fed commercial soy diets, [2] Sprague Dawley rats that received genistein by oral gavage, [3] healthy adults that consumed single servings of soy nuts, soy milk and tempeh, [4] healthy adults that chronically consumed soy milk, [5] healthy women that received 50 mg genistein orally, [6] healthy women that received 20 mg pure *S*-[-]equol orally, and 6-mo old infants fed soy infant formula and later at 3 yr fed a soy germ isoflavone supplement. The independent variable was diet and the dependent variable was plasma isoflavone levels. The outcome was that the proportion of unconjugated genistein in plasma from human adults and infants fed different soy foods, pure genistein or an isoflavone supplement was <1% at steady state and <2% at peak concentrations. In contrast, rodents fed soy diets conjugate isoflavones less efficiently, 4-30% plasma levels. Thus, there are higher circulating concentrations of biologically active unconjugated genistein in rodents.

The Field study (Field et al., 2010) determined the effect of feeding long-chain polyunsaturated fatty acids on immunity in healthy infants during the first 4 months of life. Infants of both genders [n=62] were 9.7±2.8 - 11.8±2.2 days old dependent on cohort. The independent variable was long-chain polyunsaturated fatty acids and the dependent variables were anthropometric measures, blood chemistry, experiments with pre and post culture peripheral mononuclear blood cells and cytokine production. This was a randomized trial in which lab staff were blind to all groups. The formula-fed infants were randomized at less than 14 days of age to standard term formula or formula containing long-chain polyunsaturated fatty acids. The control group was exclusively breastfed. Blood was collected at 16 weeks of age and tested for ability to proliferate and produce cytokines in response to phytohemaglutinin, beta-lactoglobulin or soy protein. Diet effects were measured by ANOVA. The outcome was that formula-fed infants who were at low risk for allergy responded differently to mitogen and food proteins *ex vivo* than infants who were breast fed. Long-chain polyunsaturated fatty acids altered some of these differences.

The Reche study (Reche et al., 2010) was a prospective open, randomized clinical study to compare the clinical tolerance of a new hydrolyzed rice formula with an extensively hydrolyzed casein formula in the feeding of infants with IgE-mediated CMA. Male and female infants [n=92] age 1.1-10.1 months were studied over a two-year period. The independent variable was hydrolyzed rice formula and the dependent variables were skin prick tests with whole cow’s milk, soy, rice and several antigens. Patients were randomized to receive extensively hydrolyzed casein formula or a new hydrolyzed rice formula with follow-up at 3, 6, 12, 18 and 24 months. The study involved measurement of growth parameters and conducting food challenge tests. The outcome was that hydrolyzed rice formula was well tolerated by infants with moderate to severe CMA suggesting that hydrolyzed rice formula is an adequate and safe alternative to cow milk protein for allergic infants. Growth rates and the development of clinical tolerance in the hydrolyzed rice formula cohort were similar to those receiving extensively hydrolyzed casein formula.

The de Mazer Papa study (de Mazer Papa et al., 2010) was a crossover, split-mouth, blinded *in situ* study to evaluate the effect of infant formula and sucrose on demineralization of tooth enamel. Test conditions included: distilled and deionized water, 10% sucrose solution, milk-based formula with/without sucrose, and SIF with/without 10% added sucrose. The independent variable was formula and the dependent variable was demineralization of enamel. The outcome was that both formulas induced significant loss of enamel, which worsened when the formula was sweetened with sucrose.

The de Mattos study (de Mattos et al., 2009) tested the hypothesis that the consumption of 3 lactose-free diets, with different degrees of complexity, would be associated with lower stool output and shorter duration of diarrhea when compared to a yogurt-based diet in the nutritional management of persistent diarrhea. The study population included 154 male or female infants and children age 1-30 months with persistent diarrhea. This was a short-term study assessing 2 semiformed/well-formed stools or no stools over a 24 hr period. The independent variable was diet [yogurt-based formula, SIF, hydrolyzed protein-based formula, amino acid-based formula] and the dependent variables were total stool output, duration of diarrhea, weight, volume of intake, failure rate or the need to change initial randomized dietary plan. The infants with persistent diarrhea randomly assigned to 1 of 4 treatment groups. Body weights and intakes of oral rehydration solution, water and formula diets were measured as well as outputs of stool, urine and vomit and recorded at 24 hr intervals. The outcome was that children fed the yogurt-based diet or amino acid-based diet had a significant reduction in stool output and duration of diarrhea.

The Hoffman study (Hoffman et al., 2008) was a double-blinded, parallel group trial testing the effect of DHA and arachidonic acid supplementation in SIF on growth and circulating fatty acid levels in healthy term infants. The study population included 244 infants, male and female, age 14-120 days. There were no data available regarding ethnicity, SES or parental education. The study period was 120 days. The independent variable was diet [SIF versus SIF containing at least 17 mg DHA and 34 mg arachidonic acid] and the dependent variables were fatty acid profiles. Participants were randomized to receive control, SIF or DHA+arachidonic acid-supplemented SIF. Anthropometric measurements were made at 14, 30, 60, 90 and 120 days of age and 24-hr dietary and tolerance recalls were recorded at 30, 60, 90 and 120 days of age. Blood samples were collected and capillary gas chromatography was used to analyze fatty acids in the red blood cell lipid fraction and in plasma phospholipids. The outcome was that the percent of fatty acids in total red blood cells and plasma phospholipids were significantly different between feeding groups at any assessed time point. Supplementation with DHA and arachidonic acid did not affect formula tolerance or the incidence of adverse side effects.

The Jirapinyo study (Jirapinyo et al., 2007) was a prospective, randomized study to determine whether chicken-based formula could replace SIF in infants with CMA. The study population included 38 infants and children age 2-24 months, male and female. The study length was 14 days. The independent variable was diet [chicken-based versus SIF] and the dependent variables were anthropometrics, blood counts and IgE tests to detect cow milk protein. The outcome was that 12 out of 18 SIF-fed infants had intolerance and could not continue with the formula. Infants, 4 out of 20, receiving chicken-based formula had evidence of clinical intolerance.

The Agustina study (Agustina et al., 2007) was a randomized, double-blind trial assessing the efficacy of an infant formula containing *Lactobacillus rhamnosus* LMG P-22799 [probiotic], inulin [prebiotic], dietary fiber [soy polysaccharides] and increased amounts of zinc and iron on the dietary management of Indonesian infants. The study population included 58 well-nourished, Indonesian, male infants age 3-12 months. The maximum treatment period was 7 days. The independent variable was diet [control versus supplemented formula]. The dependent variables were diarrhea, anthropometric measures, and blood and stool analyses. The outcome was that the duration of diarrhea was significantly shorter in the study group receiving the supplemented special formula.

The Pedrosa study (Pedrosa et al., 2006) ascertained the palatability of various formulas in a double-blind taste test. The study population included 50 healthy volunteers that were hospital workers, both male and female, with a mean age of 34.4 years [range 25-57 years]. The independent variable was formula types and the dependent variables were taste, smell and texture. The methodology involved a randomized-order, double-blind taste test with 12 milks that were evaluated for taste, smell and texture on a scale from 1-5. Statistical methods included Pearson correlation coefficients for peptide weight of reach formula versus taste score. The outcome was that soy and rice formulas had the best taste scores followed by whey, mixed and casein hydrolysates. The palatability of formulas is largely determined by the amount of bitter peptides generated by protein hydrolysis. Each of the substitution formulas exhibited statistically significant differences compared to cow’s milk in taste and texture.

The Fiocchi study (Fiocchi et al., 2006) was a double-blind, placebo-controlled food challenge prospective clinical evaluation of tolerance to hydrolyzed rice formula in children with CMA. The study population included 100 male and female infants and children age 3.17±2.93 [range 0.18-14.6 years]. The independent variable was formula type [cow milk versus rice versus hydrolyzed rice formula] and the dependent variables were allergic reaction and IgE tests. The methodology involved skin prick tests to assess allergic reactions. Sera was evaluated for specific IgE to cow’s milk protein, rice and hydrolyzed rice formula. The outcome was that 97 our of 99 subjects exhibited positive sera results to cow’s milk and or a cow milk protein fraction with the skin prick test. Subjects, 92 out of 95, exhibited positive IgE tests for cow milk or milk fractions subjects. Subjects, 21 out of 91, were positive for the IgE test for rice and 4 out of 91 were positive in response to hydrolyzed rice formula.

The Ostrom study (Ostrom et al., 2006) was a randomized, double-blind, parallel-design, two-group trial to determine if fiber-supplemented SIF reduced regurgitation in young infants. The study population included 179 infants age 13-32 days that were healthy, formula-fed, full-term infants with higher than average frequency of regurgitation. The independent variable was formula type [cow milk or soy+fiber formula] and the dependent variable was regurgitation. The methodology involved a multi-site format [6 sites] and parental reports of regurgitation. The outcome was that the initial incidence of regurgitation was similar, but after 7 days on SIF plus fiber, regurgitation was significantly lower and sustained after 28 days.

The Klemola study (Klemola et al., 2005) assessed the development of IgE antibodies specific to soy and peanuts and of allergic reactions caused by peanuts in children with CMA fed either a SIF or extensively hydrolyzed formula. The study population included 170 male and female infants age 7 months [range 2-11 months]. The independent variable was formula type [SIF or extensively hydrolyzed formula] and the dependent variables were peanut and soy IgE levels and food allergy challenge. The methodology involved diagnosis of CMA by a double-blind, placebo-controlled food challenge. Subjects were randomized to receive extensively hydrolyzed formula or SIF. Parents recorded a detailed history of peanut allergies. Soy-specific IgE was measured at time of diagnosis and ages 1, 2 and 4 yr. At age 4, peanut-specific IgE was measured. The outcome was that IgE to soy was found in 22 out of 70 children fed SIF and in 14 out of 70 fed extensively hydrolyzed formula. In an open challenge at 4 years of age, there was no immediate reaction. IgE to peanuts was found in 21 out of 70 subjects fed soy formula and 17 out of 69 fed extensively hydrolyzed formula. The use of SIF during first 2 years of life did not increase the risk of peanut-specific IgE or peanut allergy.

The Seppo study (Seppo et al., 2005) was a prospective, randomized study to compare nutrient intake, nutritional status and growth in infants with CMA who were either fed a SIF or an extensively hydrolyzed whey formula. The study population included 168 male and female infants age 7.8±2.1 months [soy cohort] and 7.5±2.2 months [extensively hydrolyzed whey formula cohort]. The independent variable was formula type and the dependent variables were anthropometric measures and blood tests. The methodology included CMA confirmation by a double-blind, placebo-controlled milk challenge, anthropometric measurements and blood analyses. Infants, 84, were fed SIF [mean start age 7.8 months] while the other 84 infants were fed extensively hydrolyzed whey formula [mean start age 7.5 months]. The outcome was that body length at 2 years of age and weight for length at 4 years of age were equivalent in both diet groups. Nutrient intake followed recommended intake in both groups. The authors noted that most infants were supplemented with calcium and vitamin D. There was greater iron inadequacy in tissue of infants in the soy group. No significant differences were observed between groups in the percent of abnormally low laboratory values [mean cell volume, hemoglobin, zinc and ferritin] or high alkaline phosphatase activity.

The Salpietro study (Salpietro et al., 2005) was a randomized clinical trial testing the efficacy and safety of a new almond-based food [almond milk] in a group of infants with CMA. The study population consisted of 52 male and female infants age 5-9 months. The independent variable was formula type and the dependent variables were anthropometric measures and CD30 serum levels. The methodology involved enrollment and randomization of infants to almond milk [n=26], SIF [n=13] or protein hydrolysate-based formula [n=13]. The study length was 6 months. The outcome was improvement in clinical symptoms and a decrease in serum levels of soluble CD30 within 5-12 days in all cases examined with no difference in growth rage, length and head circumference. The SIF and protein hydrolysate-based formulas caused secondary sensitization in some subjects [23% sensitization to soy-based].

**Supplementary Text 2**

The Fattal-Valevski study (Fattal-Valevski et al., 2009) was a follow-up study of 7 Israeli, female children, age 5-6 years, with severe epilepsy as a result of thiamine deficiency in infancy caused by a defective SIF. The findings indicated that severe infantile thiamine deficiency may result in epilepsy. The independent variable was thiamine-deficient SIF and the dependent variable was seizure activity. The methodology involved analysis of medical records of 7 children aged 5-6 years with thiamine deficiency in infancy who developed epilepsy and review of their clinical data, electroencephalography tracings, neuroimaging results, clinical course and present outcomes. The outcome was that all infants displayed seizures upon presentation (tonic, myoclonic or focal). Six infants had electroencephalography recordings at this stage and showed slow background. Five infants had no epileptic activity and one infant had focal activity. After a seizure-free period of 1-9 months, seizures recurred and all 7 children had either myoclonic or complex partial seizures. Electroencephalographs showed multifocal or generalized spike wave complexes that evolved into hypsarrhythmia in three children. The seizures were refractory to most antiepileptic drugs with four children having uncontrolled seizures. All children have mental retardation and brainstem dysfunction.

A study by Rodd and colleagues (Rodd and Mushcab, 2005) covered three case articles of infants presenting with hypocalcemic seizures during the winter months while being fed SIF. The study population included 3 infants with different ethnic backgrounds and living in different geographical regions of Eastern Canada. All subjects were male and 6 week- to 2-months old. There was no data available regarding SES or parental education level. The independent variable was SIF and the dependent variable was hypocalcemic seizures secondary to vitamin D deficiency. The methodology involved systematic examination of anthropometrics, nutrition history and blood work. The outcome was that laboratory results were consistent with vitamin D deficiency despite receiving daily reference intakes. All infants presented with hypocalcemic seizures attributable to vitamin D deficiency.

**Supplementary Text References**

Agustina R, Lukito W, Firmansyah A, Suhardjo HN, Murniati D, Bindels J (2007) The effect of early nutritional supplementation with a mixture of probiotic, prebiotic, fiber and micronutrients in infants with acute diarrhea in Indonesia. Asia Pac J Clin Nutr (Australia) 16:435-442.

de Mattos AP, Ribeiro TC, Mendes PS, Valois SS, Mendes CM, Ribeiro HC,Jr (2009) Comparison of yogurt, soybean, casein, and amino acid-based diets in children with persistent diarrhea. Nutr Res (United States) 29:462-469.

de Mazer Papa AM, Tabchoury CP, Del Bel Cury AA, Tenuta LM, Arthur RA, Cury JA (2010) Effect of milk and soy-based infant formulas on in situ demineralization of human primary enamel. Pediatr Dent (United States) 32:35-40.

Fattal-Valevski A, Bloch-Mimouni A, Kivity S, Heyman E, Brezner A, Strausberg R, Inbar D, Kramer U, Goldberg-Stern H (2009) Epilepsy in children with infantile thiamine deficiency. Neurology (United States) 73:828-833.

Field CJ, Van Aerde JE, Goruk S, Clandinin MT (2010) Effect of feeding a formula supplemented with long-chain polyunsaturated fatty acids for 14 weeks improves the ex vivo response to a mitogen and reduces the response to a soy protein in infants at low risk for allergy. J Pediatr Gastroenterol Nutr (United States) 50:661-669.

Fiocchi A, Restani P, Bernardini R, Lucarelli S, Lombardi G, Magazzu G, Marseglia GL, Pittschieler K, Tripodi S, Troncone R, Ranzini C (2006) A hydrolysed rice-based formula is tolerated by children with cow's milk allergy: A multi-centre study. Clin Exp Allergy (England) 36:311-316.

Hoffman D, Ziegler E, Mitmesser SH, Harris CL, Diersen-Schade DA (2008) Soy-based infant formula supplemented with DHA and ARA supports growth and increases circulating levels of these fatty acids in infants. Lipids (United States) 43:29-35.

Jirapinyo P, Densupsoontorn N, Wongarn R, Thamonsiri N (2007) Comparisons of a chicken-based formula with soy-based formula in infants with cow milk allergy. Asia Pac J Clin Nutr (Australia) 16:711-715.

Klemola T, Kalimo K, Poussa T, Juntunen-Backman K, Korpela R, Valovirta E, Vanto T (2005) Feeding a soy formula to children with cow's milk allergy: The development of immunoglobulin E-mediated allergy to soy and peanuts. Pediatr Allergy Immunol (England) 16:641-646.

Lasekan J, Baggs G, Acosta S, Mackey A (2015) Soy protein-based infant formulas with supplemental fructooligosaccharides: Gastrointestinal tolerance and hydration status in newborn infants. Nutrients (Switzerland) 7:3022-3037.

Ostrom KM, Jacobs JR, Merritt RJ, Murray RD (2006) Decreased regurgitation with a soy formula containing added soy fiber. Clin Pediatr (Phila) 45:29-36.

Pedrosa M, Pascual CY, Larco JI, Esteban MM (2006) Palatability of hydrolysates and other substitution formulas for cow's milk-allergic children: A comparative study of taste, smell, and texture evaluated by healthy volunteers. J Investig Allergol Clin Immunol (Spain) 16:351-356.

Reche M, Pascual C, Fiandor A, Polanco I, Rivero-Urgell M, Chifre R, Johnston S, Martin-Esteban M (2010) The effect of a partially hydrolysed formula based on rice protein in the treatment of infants with cow's milk protein allergy. Pediatr Allergy Immunol (England) 21:577-585.

Rodd C, Mushcab SA (2005) Hypocalcemic seizures secondary to nutritional vitamin D deficiency in 3 infants fed soy formula. Clin Pediatr (Phila) (United States) 44:455-457.

Salpietro CD, Gangemi S, Briuglia S, Meo A, Merlino MV, Muscolino G, Bisignano G, Trombetta D, Saija A (2005) The almond milk: A new approach to the management of cow-milk allergy/intolerance in infants. Minerva Pediatr (Italy) 57:173-180.

Seppo L, Korpela R, Lonnerdal B, Metsaniitty L, Juntunen-Backman K, Klemola T, Paganus A, Vanto T (2005) A follow-up study of nutrient intake, nutritional status, and growth in infants with cow milk allergy fed either a soy formula or an extensively hydrolyzed whey formula. Am J Clin Nutr (United States) 82:140-145.

Setchell KD, Brown NM, Zhao X, Lindley SL, Heubi JE, King EC, Messina MJ (2011) Soy isoflavone phase II metabolism differs between rodents and humans: Implications for the effect on breast cancer risk. Am J Clin Nutr (United States) 94:1284-1294.

Skau JK, Bunthang T, Chamnan C, Wieringa FT, Dijkhuizen MA, Roos N, Ferguson EL (2014) The use of linear programming to determine whether a formulated complementary food product can ensure adequate nutrients for 6- to 11-month-old Cambodian infants. Am J Clin Nutr (United States) 99:130-138.

# Supplementary Figure 1

**Legend**: Food labels from Enfamil Premium and ProSobee powder infant formulas. Enfamil Premium was the leading powder baby formula in the United States in 2012. ProSobee is the Enfamil SIF product.

**Supplementary Figure 2**


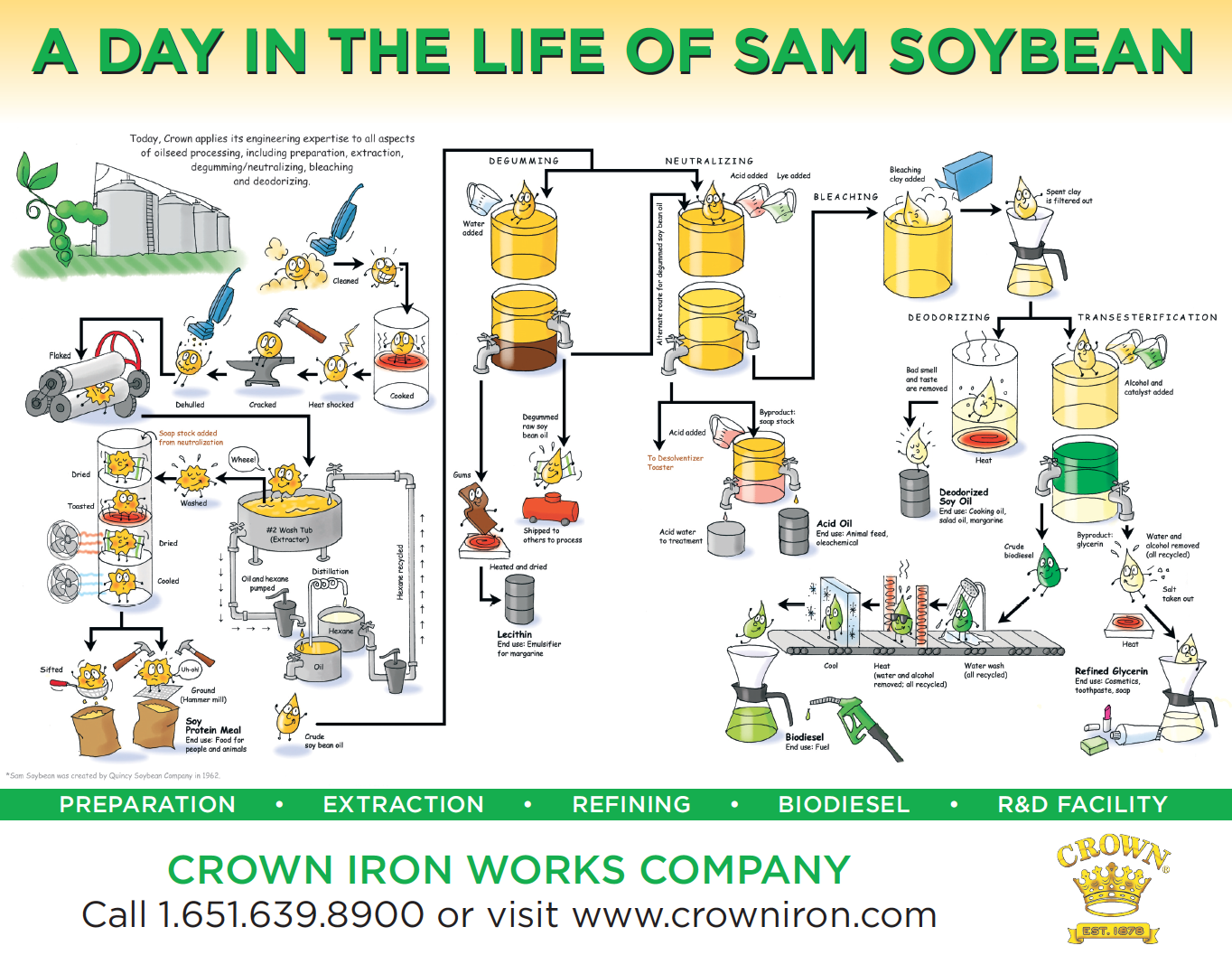


**Legend**: A day in the Life of Sam Soybean reproduced with permission from the Crown Iron Works Company.

| **Supplementary Table 1: Seizures in Probands Not Associated with Maternal Autoimmunity.** | | | | |
| --- | --- | --- | --- | --- |
| **Cohort** | **N^a^** | **% Exhibiting Seizures** | ***P*^b^** | **CI^b^** |
| **Females** | | | | |
| Mom AID^d^ | 7 | 0 | 1.0 | 0 (0-13) |
| Mom no AID | 280 | 6.1 |  |  |
| **Males** | | | | |
| Mom AID | 42 | 2.4 | 1.0 | 0.53 (0.027-3.6) |
| Mom no AID | 1790 | 4.4 |  |  |
| **Genders Combined** | | | | |
| Mom AID | 49 | 2.0 | 0.73 | 0.43 (0.022-2.9) |
| Mom no AID | 2070 | 4.6 |  |  |
| ^a^N is the number of subjects per cohort.  ^b^P values were calculated by Fisher’s exact test (two-tail).  ^c^Odds ratios are presented with 95% confidence intervals.  ^d^Autoimmune Disorder abbreviated AID. | | | | |

| **Supplementary Table 2: Use of Soy-Based Infant Formula Is Dependent on Maternal Autoimmune Status.** | | | | |
| --- | --- | --- | --- | --- |
| **Cohort** | **N^a^** | **% Mothers with**  **AID** | ***P*^b^** | **CI^b^** |
| **Females** | | | | |
| Soy | 41 | 4.9 | 0.03 | 2.3 (1.1-4.7) |
| Non-Soy | 217 | 1.4 |  |  |
| **Males** | | | | |
| Soy | 267 | 4.5 | 0.18 | 3.7 (0.41-28) |
| Non-Soy | 1385 | 2.0 |  |  |
| **Genders Combined** | | | | |
| Soy | 308 | 4.5 | 0.01 | 2.4 (1.2-4.8) |
| Non-Soy | 1602 | 1.9 |  |  |
| ^a^N is the number of subjects per cohort.  ^b^P values were calculated by Fisher’s exact test (two-tail).  ^c^Odds ratios are presented with 95% confidence intervals.  ^d^Autoimmune Disorder abbreviated AID. | | | | |

| **Supplementary Table 3: Ethnicity of SFARI Dataset** | | | | | |
| --- | --- | --- | --- | --- | --- |
| **Cohort** | **N** | **% White** | **% Asian** | **% African**  **American** | **% Other** |
| Females + Soy | 44 | 77.3 | 4.5 | 9.1 | 9.1 |
| Females No Soy | 217 | 82.5 | 3.2 | 3.2 | 11.1 |
| Males + Soy | 297 | 79.1 | 2.4 | 5.1 | 13.5 |
| Males No Soy | 1391 | 78.6 | 4.2 | 3.4 | 13.8 |
